# CircSEC24B activates autophagy and induces chemoresistance of colorectal cancer via OTUB1-mediated deubiquitination of SRPX2

**DOI:** 10.1038/s41419-024-07057-y

**Published:** 2024-09-27

**Authors:** Di Wang, Yongge Li, Weilong Chang, Meina Feng, Yiming Yang, Xiuxiang Zhu, Zhibo Liu, Yang Fu

**Affiliations:** 1 Department of Gastrointestinal Surgery, The First Affiliated Hospital of Zhengzhou University, Zhengzhou, China; 2 Department of Cardiology, The First Affiliated Hospital of Zhengzhou University, Zhengzhou, China; 3 Department of Neurology, Wuhan Brain Hospital, General Hospital of the YANGTZE River Shipping, Wuhan, China; 4 Department of General Surgery, Ruijin-Hainan Hospital, Shanghai Jiao Tong University School of Medicine, Hainan, China; 5 Department of Gastrointestinal Surgery, Union Hospital, Tongji Medical College, Huazhong University of Science and Technology, Wuhan, China

**Keywords:** Colorectal cancer, Cell invasion

## Abstract

Circular RNAs (circRNAs) are a type of regulatory RNA that feature covalently closed single-stranded loops. Evidence suggested that circRNAs play important roles in the progression and development of various cancers. However, the impact of circRNA on autophagy-mediated progression of colorectal cancer (CRC) remains unclear. The objective of this project was to investigate the influence of circSEC24B on autophagy and its underlying mechanisms in CRC. To validate the presence and circular structure of circSEC24B in CRC cells and tissues, PCR and Sanger sequencing techniques were employed. Drug resistance and invasive phenotype of CRC cells were evaluated using CCK8, transwell, and Edu assays. Gain- and loss-of-function experiments were conducted to assess the effects of circSEC24B and its protein partner on the growth, invasion, and metastasis of CRC cells in vitro and in vivo. Interactions between circSEC24B, OTUB1, and SRPX2 were analyzed through immunofluorescence, RNA-pulldown, and RIP assays. Mass spectrometry analysis was used to identify potential binding proteins of circRNA in CRC cells. Vectors were constructed to investigate the specific structural domain of the deubiquitinating enzyme OTUB1 that binds to circSEC24B. Results showed that circSEC24B expression was increased in CRC tissues and cell lines, and it enhanced CRC cell proliferation and autophagy levels. Mechanistically, circSEC24B promoted CRC cell proliferation by regulating the protein stability of SRPX2. Specifically, circSEC24B acted as a scaffold, facilitating the binding of OTUB1 to SRPX2 and thereby enhancing its protein stability. Additionally, evidence suggested that OTUB1 regulated SRPX2 expression through an acetylation-dependent mechanism. In conclusion, this study demonstrated that circSEC24B activated autophagy and induced chemoresistance in CRC by promoting the deubiquitination of SRPX2, mediated by the deubiquitinating enzyme OTUB1.

## Introduction

Colorectal cancer (CRC) is the third most prevalent carcinoma diagnosed worldwide and the fourth highest cause of cancer-associated death globally, with a projected increase of 60% in its burden by 2030 [1]. Alarmingly, 25–30% CRC patients are diagnosed in stage IV, indicative of distant metastases, for which there is currently no definitive cure, whereas almost all stage I CRC patients can be successfully treated [2]. Currently, management strategies of CRC mainly contains surgery, immunotherapy, chemotherapy, and radiotherapy [3, 4]. Advanced CRC patients are often treated with the chemotherapy drug 5-fluorouracil (5FU), either alone or in combination with oxaliplatin (OXA) and other targeted therapies [5]. Nevertheless, these patients frequently experience cancer recurrence due to drug resistance, resulting in a dismal 5-year survival rate of less than 10% [6]. Thus, it is critical to illuminate the potential mechanism of chemoresistance for the development of new strategies to overcome drug resistance of advanced CRC patients.

Autophagy, a vital cellular response to adverse conditions such as nutrient deprivation, drug toxicity, and hypoxia, has garnered significant attention in recent years [7, 8]. Various studies showed that autophagy was related to cancer resistance to a lot of chemotherapeutic drugs such as 5FU, cisplatin, and doxorubicin [9–11]. Emerging evidence suggests that autophagy has various roles in tumor progression [12] and that autophagy induced by therapeutic intervention represent a novel mechanism of drug resistance to chemotherapy [13]. Circular RNAs (circRNA), a group of regulatory RNA featured by covalently closed single-stranded loops, are produced by precursor mRNA back-splicing of exons [14–16]. Accumulating evidence indicated that circRNAs are associated with the development and pathogenesis of a variety of carcinomas [17, 18]. In addition, by regulating the expression of autophagy-associated genes, circRNA played a critical role in modulating autophagy [19, 20], thereby attracting growing interest in their potential role in carcinoma treatment as well as drug resistance. Moreover, autophagy has been shown to be intimately involved in CRC progression. For instance, Wei et al. suggested that FAT4 can regulate the EMT and autophagy in CRC cells, partly via the PI3K-AKT signaling axis [21]. Jin et al. indicated that YAP inhibited autophagy and promoted progression of CRC by upregulating Bcl-2 expression [22]. However, whether circRNAs are involved in autophagy-mediated drug resistance in CRC is still unclear.

Various studies suggested that SRPX2 played a critical role in the development of CRC. For instance, Zhou et al. indicated that SRPX2 regulated colon cancer cell metabolism through miR-192/215 via the PI3K-Akt axis [23]. Øster et al. reported that Non-CpG island promoter hypomethylation and miR-149 regulate the expression of SRPX2 in CRC [24]. Meanwhile, a few study found that OTUB1 had a critical effect on CRC progression. For example, Zhou et al. revealed that OTUB1 promoted metastasis and functioned as a biomarker of poor prognosis in CRC [25]. Yuan et al. found that miR-542-3p suppressed CRC cell proliferation, migration as well as invasion via targeting OTUB1 [26]. Nevertheless, the potential association between SRPX2, OTUB1, circRNAs, and autophagy-mediated CRC resistance has yet to be reported.

In the present study, we aimed to unravel the mechanism by which circSEC24B activated autophagy and induced chemoresistance in CRC. Our findings may provide novel insights into CRC development and facilitate the identification of autophagy-associated treatment targets for CRC.

## Materials and methods

### Bioinformatics data analyzing patient tissue

We applied Robust Multi-Array Average and Linear Models for Microarray (LIMMA) algorithm to normalize the circRNA raw count expression matrix [27]. The differentially expressed circRNA/proteins between the experiment group and NC group were screened out using ‘limma’ package [27]. The inclusion criteria were set as *P* < 0.05 and logFC > 1.3.

Sixty-eight para-tumor and CRC tissues were collected from the Union Hospital affiliated to the Tongji Medical College of Huazhong University of Science and Technology, Wuhan, China (Table S1). The diagnosis of CRC patients was confirmed by the histopathological reports. All procedures were on the basis of guidelines that were put forth by the Declaration of Helsinki. All patients signed an informed consent form prior to participation in the trial and the protocols were approved by the ethics committee of the Huazhong University of Science and Technology.

### Cell culture

Human normal colonic mucosal epithelial cells (NCM460) and human CRC cell lines, including HCT116, SW48, LoVo, DLD-1, SW620, as well as HEK-293T cells were purchased from Procell (Wuhan, China) and these cells were maintained in DMEM containing 10% fetal bovine serum with 100 U/ml penicillin/streptomycin in an incubator at 37 °C with 5% CO_2_.

To construct oxaliplatin-resistant resistant CRC cells, HCT116 and LoVo cells were first exposed to medium containing OXA with an initial concentration of 1 μmol/L. Subsequently, the concentration of OXA was gradually increased, and the cells were continuously cultured under these conditions until they became stably resistant. The resistance of the constructed cell lines is confirmed by evaluating their viability, proliferation rate, and dose-response curves in the presence of OXA (exposed to 25 μM for HCT116, 20 μM for LoVo).

### Cell transfection

The circSEC24B overexpression plasmid (circSEC24B-OE) was synthesized by inserting the full-length sequence of circSEC24B into the pLO5-ciR lentiviral vector (Geneseed Biotech Co., Ltd). An shRNA lentiviral plasmid specifically designed to knock down circSEC24B expression (sh-circSEC24B) was generated by inserting annealed shRNA template sequence into the pLKO.1 vector. The pcDNA (Vector) and pcDNA-OTUB1/SRPX2 overexpression (OTUB1-OE/SRPX2-OE) plasmids were obtained from GenePharma (Shanghai, China). Autophagic flux in CRC cells was monitored using the mRFP-GFP-LC3 adenovirus (Hanbio Co. Ltd., Shanghai, China). The transfections were performed using Lipofectamine 3000 reagent (Thermo Fisher Scientific) for 48 h in accordance to the manufacturer’s protocol. The infection efficiency was assessed via RT-qPCR analysis.

### Quantitative reverse transcription-polymerase chain reaction (RT-qPCR)

Total RNAs were collected from cells using the TRIzol reagent (Thermo Fisher, Shanghai, China) according to the manufacturer’s instructions. Then, reverse transcription was conducted via reverse transcription kit (Promega). After that, RT-qPCR was conducted using a SYBR Premix Ex Taq (TaKaRa) kit on the basis of an AB 7500 Real-time PCR system. The gene-specific primer sets were used at a final concentration of 0.5 µM. All RT-qPCR assays were conducted three times. mRNA expression levels were normalized to GAPDH, the relative gene expression was quantified via the 2^−ΔΔCt^ method. All primer sequences used in our study are listed in Table S2.

### Western blot

Proteins were extracted with RIPA buffer (Beyotime, Shanghai, China) and quantified using bicinchoninic acid assay. The proteins were then separated by electrophoresis and transferred onto a polyvinylidene fluoride membrane (Millipore, USA). Afterward, the membranes were blocked with 5% skim milk for 1 h at room temperature. Next, the samples were incubated overnight at 4 °C with primary antibodies diluted in appropriate buffers: (PSMD7, 1:5000, 68188-1-Ig, Proteintech; EIF3F, 1:1000, A7023, Abclonal; GAPDH, 1:5000, 10494-1-AP, Proteintech; LC3 II, 1:2000, ab192890, Abcam; SQSTM1, 1:10,000, ab109012, Abcam; DLG4, 1:4000, 20665-1-AP, Proteintech; SGK1, 1:1000, 28454-1-AP, Proteintech; IDO1, 1:1000, 13268-1-AP, Proteintech; SRPX2, 1:1000, 66266-1-Ig, Proteintech; FCAN, 1:20,000, 10205-2-AP, Proteintech; E-cadherin, 1:20,000, 20874-1-AP, Proteintech; N-cadherin, 1:4000, 22018-1-AP, Proteintech; p-FAK, 1:1000, ab81298, Abcam; FAK, 1:5000, 66258-1-Ig, Proteintech; p-SRC, 1:2000, ab40660, Abcam; SRC, 1:500, 11097-1-AP, Proteintech; OTUB1, 1:1000, PA5-118134, ThermoFisher; SIRT1, 1:1000, # 8469, Cell Signaling; Rabbit polyclonal anti-Acetylated-Lysine, 1:1000, t# 9441, Cell Signaling). Following primary antibody incubation, the membranes were washed and incubated with the corresponding secondary antibody for 1.5 h at room temperature. Finally, the proteins were visualized using electrochemiluminescence, a standard method for detecting antibody binding on membranes.

### Cell counting kit-8 (CCK-8) assay

CRC cell viability was detected via conducting CCK-8 assay (Dojindo, Japan). Following transfection, CRC cells were added into a 96-well plate at a density of 2 × 10^3^ cells/well, and cultured for 24 h in a 37 °C incubator. At 24, 48, and 72 h post seeding, 10 μL of CCK-8 solution was added to each well, and the cells were further incubated for 2 h at 37 °C. The optical density values of each well were measured at 450 nm with a microplate reader (Thermo, USA).

### 5‑ethynyl‑20‑deoxyuridine (EdU) analysis

The proliferation capacity of CRC cell was assessed with the EdU assay kit (Ribobio, Guangzhou, China). Briefly, CRC cells were cultured into 96-well plates at a density of 1 × 10^4^ cells/well for 20 h. Subsequently, 50 μM EdU reagent was added to each well with culture medium at 37 °C, 5% CO_2_ for 2 h. Following incubation, the cells were fixed with 4% formaldehyde for 30 min. The nuclei were then stained with Hoechst 33342 for 15 min. Eventually, the fluorescence images were captured using a fluorescence microscope (Olympus, Tokyo, Japan).

### Colony formation assay

The transfected CRC cells were seeded into the 6-well plates at a density of 500 cells per well and incubated at 37 °C for 2 weeks. Following this, the cells were fixed by using 10% formaldehyde at 37 °C. Subsequently, the cells were stained using 0.5% crystal violet solution for 15 min. The images were taken, and the number of colonies more than 50 cells were counted with a microscope.

### Coimmunoprecipitation assay

Coimmunoprecipitation assay was conducted as previously described [28]. Primary antibodies specific for OTUB1 (1:1000, PA5-118134, ThermoFisher) and FLAG (1:30, ab205606, Abcam) were used.

### RNA-fluorescence in situ hybridization (RNA -FISH)

RNA-FISH was carried out employing the Fluorescent In Situ Hybridization Kit (RiboBio) on the basis of the manufacturer’s instructions. Biotin-labeled probes that targeted circSEC24B (Table S2) were offered by RiboBio. Fluorescent images were obtained with a confocal laser scanning microscope (Olympus).

### Biotin-labeled RNA pull-down

RNA pull-down assays were performed with a magnetic RNA-protein pulldown kit (Thermo, Waltham, MA, USA) in accordance with the manufacturer’s protocol. The RNA-bound proteins were assessed using mass spectrometry and western blotting assay.

### RNA immunoprecipitation (RIP)

RIP assays were performed by using a specific RNA-binding protein

immunoprecipitation kit (Geneseed, Guangzhou, China) according to the manufacturer’s instructions. Co-precipitated RNA was evaluated via RT-qPCR assays with specific primers.

### Chromatin immunoprecipitation (ChIP) assay

ChIP analysis was conducted as described in the previous study [29]. IgG was used as negative control, anti-RNA Polymerase II was adopted as positive control and ZFX antibody (ZFX, 1:100, PA5-78234, ThermoFisher) was used for immunoprecipitation of chromatin.

### Immunofluorescence (IF) assay

The IF assay was performed as described previously [30]. Fluorescence images were acquired using a fluorescence microscope. IHC analysis was performed as described previously [31].

### Immunohistochemistry (IHC) assay and histology assay

H&E and IHC assays of Tumor tissues were performed as described previously [32]. Primary antibody for Ki-67 (1:5,000, ProteinTech) was applied. All images were obtained by using an Olympus BX-51 light microscope.

### Animal experimentation

Animal experiments were carried out with BALB/c nude mice (male, 6 weeks old). The stably transfected HCT116/OXA cells (1 × 10^6^ cancer cells every mouse, *n* = 5 each group) or mock vector were subcutaneously injected into the left flanks of nude mice. Tumor growth was assessed every 5 days, and tumor volumes were calculated Tumor volumes were calculated as length × width^2^ × 0.5. After 4 weeks, mice were sacrificed, and the tumor weights were calculated. All animal experiments were carried out according to the guidelines and experiments were approved by the Institutional Animal Care Use Committee of Huazhong University of Science and Technology.

### Statistical analysis

All data are presented as Means ± SEM. Statistical significance was assessed using Student’s *t* test for two cohorts, or by one-way ANOVA for three or more groups using GraphPad Prism Seven software. *p* < 0.05 was considered to be significant.

## Results

### CircSEC24B is upregulated in CRC cells and tissues

**Fig. 1:**
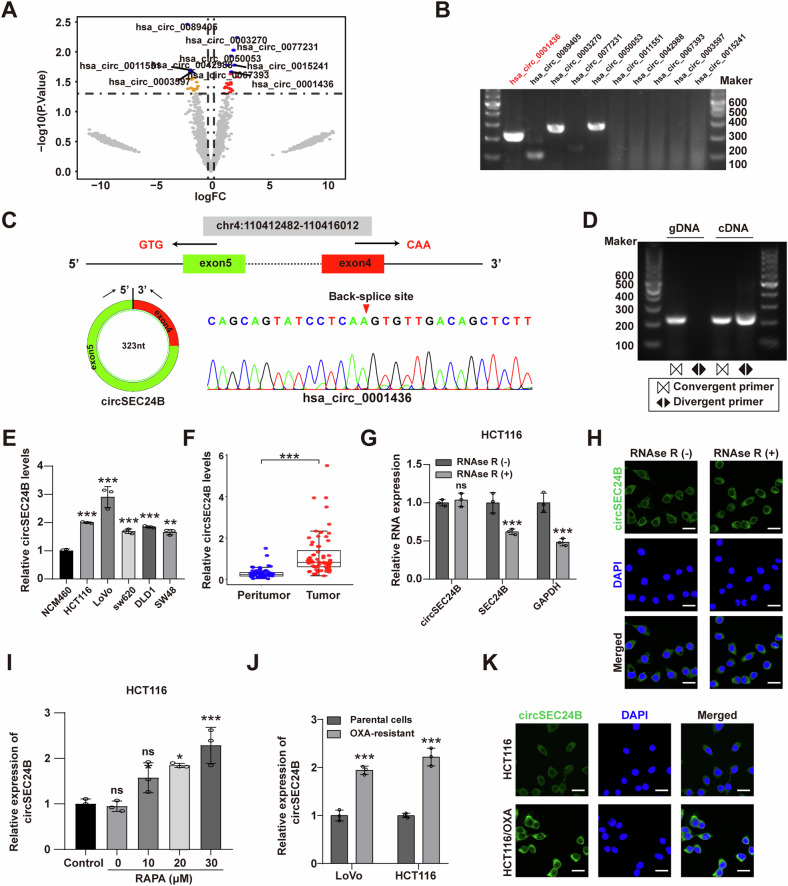
CircSEC24B was upregulated in CRC cells and tissues. **A** Top 10 differentially expressed circRNAs between RAPA-treated and NC group were visualized by Volcano plot. **B** RT-PCR assay for the top 10 circRNAs with divergent primers. **C** The backsplice junction site of circSEC24B was determined via Sanger sequencing. **D** RT-PCR assay with cDNA and genomic DNA using circSEC24B divergent and convergent primers. **E** RT-qPCR assays for circSEC24B with the NCM460 and the five CRC cell lines. **F** RT-qPCR assays for circSEC24B by CRC tissue and normal tissue samples. **G** RT-qPCR assay for circSEC24B, SEC24B GAPDH with or without RNAse R treatment. **H** RNA-FISH for circSEC24B with or without treatment of RNAse R, DAPI (blue) was used for nuclei staining. **I** RT-qPCR assay for circSEC24B expression with various dose of RAPA in HCT116 cell line. **J** RT-qPCR assay for circSEC24B in OXA-resistant or OXA-nonresistant LoVo and HCT116 cell lines. **K** RNA-FISH assay for circSEC24B in HCT116 and HCT116/OXA cell lines.

To identify key biological pathways in resistant CRC cells, we performed GSEA analysis, revealing significant enrichment of autophagy-related pathways (Fig. S1A). To explore the circRNAs that play important roles in autophagy-mediated OXA resistance of CRC cells, we treated HCT116/OXA cells with autophagy agonist rapamycin (RAPA) and subjected samples to circRNA sequencing. Among the top ten differentially expressed circRNAs, three were confirmed by DNA electrophoresis (Fig. 1A, B) and Sanger sequencing (Figs. 1C, D and S1B–E). RT-qPCR analysis of these circRNAs in normal colonic epithelial cells (NCM460), CRC cell lines, and CRC tissues revealed that only hsa_circ_0001436 (designated circSEC24B) was consistently upregulated in CRC cells and tissues (Figs. 1E, F and S1F–I). Notably, circSEC24B was most significantly upregulated in HCT116 and LoVo cells, which were chosen for further study. The resistance to digestion by RNase R exonuclease suggested a circular RNA structure for circSEC24B (Figs. 1G, H and S1J, K). Then we investigated whether circSEC24B was involved in autophagy-related CRC drug resistance and found that the expression of circSEC24B was significantly enhanced after treatment with rapamycin in a dose-dependent manner (Figs. 1I and S1L). Meanwhile, the expression of circSEC24B was significantly upregulated in OXA-resistant CRC cells compared to that in non-resistant cells (Fig. 1J). FISH assay showed cytoplasmic enrichment of circSEC24B in CRC cells (Figs. 1K and S1M).

### CircSEC24B promoted CRC cell proliferation and drug resistance

**Fig. 2:**
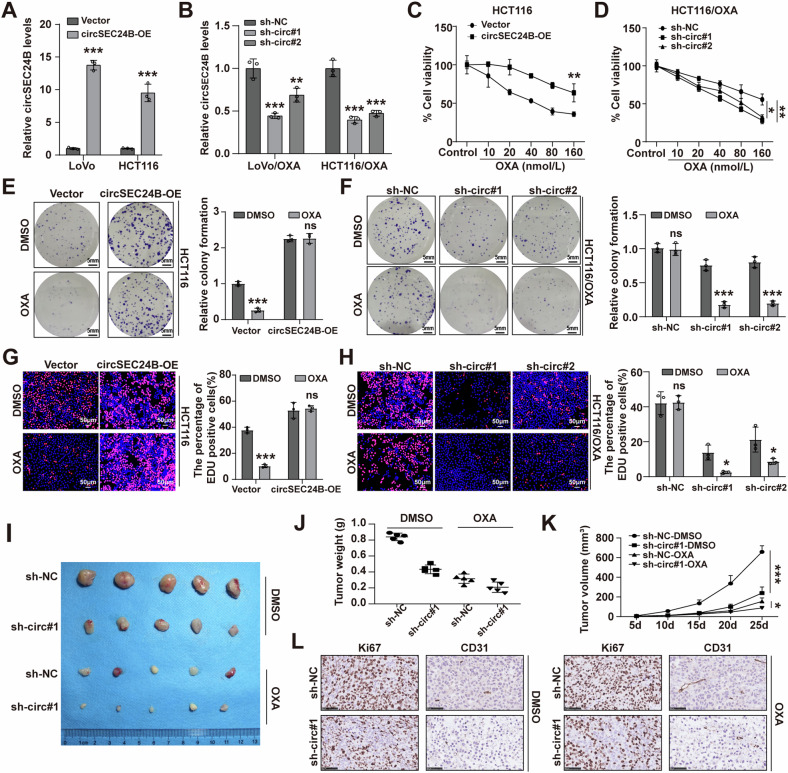
CircSEC24B promoted CRC cell proliferation and OXA resistance. **A** RT-qPCR validation of circSEC24B expression in LoVo and HCT116 CRC cell lines transfected with circSEC24B overexpression vectors. **B** RT-qPCR validation of circSEC24B expression in OXA-resistant LoVo/OXA and HCT116/OXA CRC cell lines transfected with sh-circSEC24B. **C**, **D** HCT116 and HCT116/OXA cell viability was assessed with circSEC24B overexpression or knockdown in different dose of OXA. **E**, **F** HCT116, and HCT116/OXA cell proliferation ability was assessed through colony formation assay with circSEC24B overexpression or knockdown. **G**, **H** HCT116 and HCT116/OXA cell proliferation ability was assessed through EDU assay with circSEC24B overexpression or knockdown. **I** The effect of OXA and knockdown of circSEC24B on xenografts formed by subcutaneous injection of the HCT116/OXA cell line. **J**, **K** The effect of OXA and knockdown of circSEC24B on tumor weight and tumor volume of xenografts formed by subcutaneous injection of the HCT116/OXA cell line. **L** Immunohistochemistry assays for Ki-67 and CD31 by xenografts formed by subcutaneous injection of the HCT116/OXA cell line with NC or sh-circSEC24B.

To evaluate the role of circSEC24B in the CRC cells, we transfected CRC cells with circSEC24B overexpression plasmid and shRNA. The results showed that we constructed the expression vector successfully (Fig. 2A, B). After upregulation of circSEC24B, the proliferative capacity of CRC cells was significantly increased, while downregulation of circSEC24B had the opposite effect in both selected CRC cell lines with or without OXA treatment (Figs. 2C–H and S2A–F). Rescue assays demonstrated that circSEC24B knockdown blocked proliferative ability, which was reversed by circSEC24B overexpression (Fig. S2G–L). In vivo, sh-circSEC24B reduced tumor growth and weight in nude mice injected with HCT116/OXA cells (Fig. 2I–K). IHC analysis suggested downregulation of Ki-67 and CD31 upon circSEC24B knockdown (Fig. 2L). Collectively, these results show that circSEC24B promotes CRC cell proliferation and drug resistance.

### CircSEC24B elevated the autophagy level of CRC cells

**Fig. 3:**
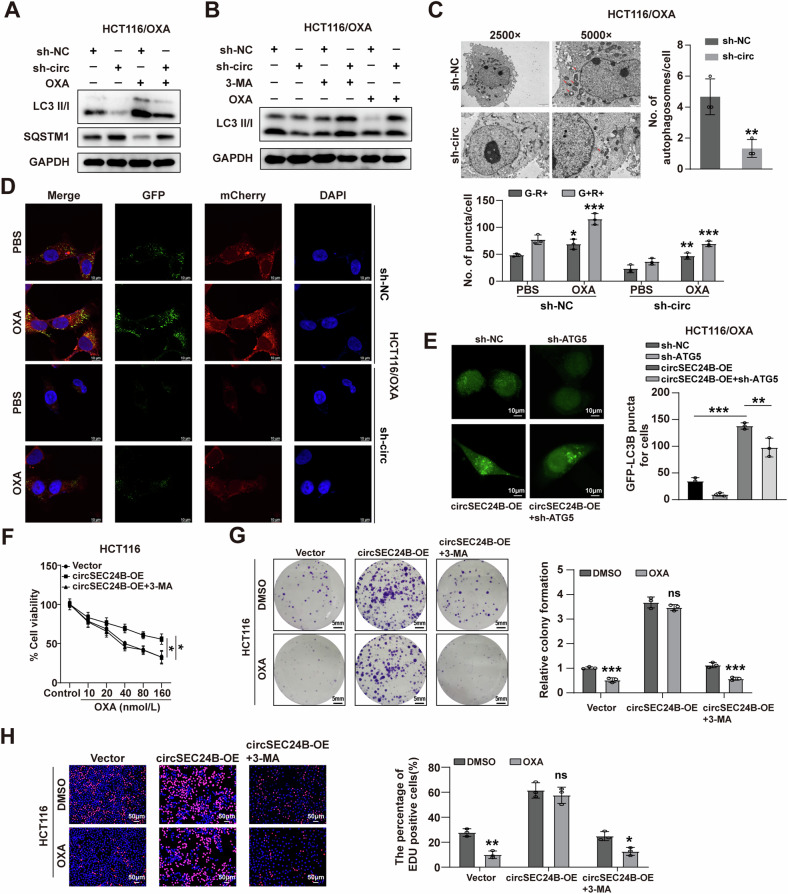
CircSEC24B elevated the autophagy level of CRC cells. **A** Western blotting assay for the expression levels of LC3-II and p62 with or without OXA treatment in the NC and sh-circSEC24B-transfected HCT116/OXA cells. **B** Western blotting assay for the expression levels of LC3-II with or without OXA, 3-MA treatment in NC or sh-circSEC24B-transfected HCT116/OXA cells. **C** Representative TEM images of the HCT116/OXA cells. The red arrows indicated the autophagosomes in the cytoplasm. The graph on the right summarized the numbers of autophagosomes in the cytoplasm. **D** Representative autophagy puncta images of the NC and sh-circSEC24B-transfected cells with or without OXA treatment. **E** GFP-LC3-II puncta formation in circSEC24B-OE or scrambled HCT116/OXA cells transfected with or without ATG5 shRNA. The effects of circSEC24B-overexpression with or without OXA and 3-MA treatment on cell viability and proliferation ability were assessed by CCK-8 (**F**), colony formation (**G**), and EDU assays (**H**).

Since the expression level of circSEC24B was positively correlated with the treatment concentration of RAPA, we assessed autophagy markers LC3-I, LC3-II, SQSTM1, ATG5, and Beclin1 by western blotting. circSEC24B downregulation and 3-MA (early-stage autophagy inhibitor) decreased LC3-II, ATG5, and Beclin1 levels while increasing SQSTM1, with the opposite effect observed upon upregulation (Figs. 3A, B and S3A–D, G). Baf-1 (late-stage autophagy inhibitor) treatment increased both LC3-II and p62 levels (Fig. S3C). Similarly, transmission electron microscopy (TEM) images revealed a smaller number of autophagosomes in the cytoplasm of the drug-resistant cells in sh-circSEC24B group compared to the NC group (Figs. 3C and S3E). mCherry-GFP-LC3 reporter assay showed that increasing number of G+R+ and G−R+ puncta induced by OXA treatment could be reversed by sh-circSEC24B transfection (Figs. 3D and S3F). Notably, elevated expression of LC3-II caused by circSEC24B overexpression could be blocked by knockdown of ATG5, suggesting that circSEC24B facilitated activated autophagy in CRC cells (Fig. 3E). Next, we explored whether the cancer-promoting effects of circSEC24B were influenced by 3-MA. As expected, circSEC24B overexpression enhanced HCT116 and LoVo cell viability and proliferation, which can be reversed by 3-MA (Figs. 3F–H and S3H–J). Taken together, circSEC24B promoted autophagy and drug resistance in CRC cells.

### CircSEC24B promoted the expression of SRPX2 in CRC cells

**Fig. 4:**
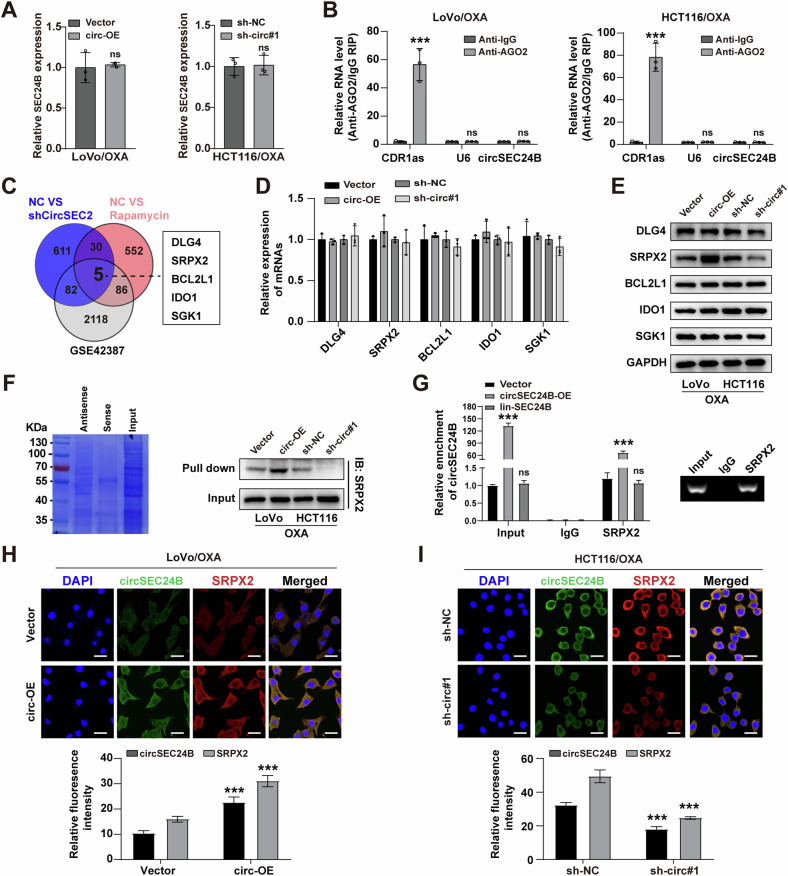
CircSEC24B promoted the expression of SRPX2 in CRC cells. **A** RT-qPCR assay for SEC24B mRNA with a stable transfection of circSEC24B or sh-circSEC24B in LoVo/OXA and HCT116/OXA cells. **B** The argonaute 2 (Ago2) cross-linking immunoprecipitation to assess the correlation between circSEC24B and AGO2 in LoVo/OXA and HCT116/OXA cells. **C** The differentially expressed proteins induced by shCircSEC24B or RAPA were intersected with DEGs of GSE42387 sets. **D** RT-qPCR assay for the mRNA expression level of the selected five genes with circSEC24B overexpression or knockdown. **E** Western blot assay for DLG4, SRPX2, BCL2L1, SGK1, and ABTB2 with upregulation or downregulation of circSEC24B in LoVo/OXA and HCT116/OXA cells. **F** Biotin-labeled RNA pull-down assay for evaluation of the interplay between circSEC24B and SRPX2 in LoVo/OXA and HCT116/OXA cell line. **G** RIP assay was used to assess the endogenous enrichment of circSEC24B with SRPX2 antibody in HCT116/OXA cells. **H**, **I** Dual RNA-FISH and immunofluorescence assays to the localization of circSEC24B and SRPX2 with upregulation or downregulation of circSEC24B in LoVo/OXA and HCT116/OXA cells.

To analyze the potential mechanisms underlying the cancer-promoting effect of circSEC24B, we explored the impacts of circSEC24B on the expression of its parent gene SEC24B. Stable transfection of circSEC24B or sh-circSEC24B did not alter the mRNA levels of SEC24B in CRC cells (Fig. 4A), indicating that circSEC24B did not exert a cis-regulatory effect on the maternal SEC24B gene. Next, we further investigated whether circSEC24B severed as a miRNA sponge, the argonaute 2 (Ago2) cross-linking immunoprecipitation was performed, and the result showed that circSEC24B did not bind to Ago2, which excluded the possibility of circSEC24B acting through the ceRNA network mechanism (Fig. 4B). Next, we overlapped the differentially expressed genes (DEGs) in the sh-circSEC24B group compared with the NC group, as well as in the RAPA group compared with the NC group, with DEGs in dataset GSE42387. The intersection result identified five potential circSEC24B targets (Fig. 4C). RT-qPCR assay was conducted to evaluate the mRNA expression level of these five genes, and the results showed that upregulation or downregulation of circSEC24B did not change the mRNA levels of the five genes (Fig. 4D). However, the result of western blot assay revealed that upregulation or downregulation of circSEC24B promoted or inhibited the expression of SRPX2, respectively, in CRC cells, but not the other four genes (Fig. 4E). Then, biotin-labeled RNA pull-down assay was performed to assess the interaction between circSEC24B and SRPX2. The result showed that SRPX2 protein was pulled down by circSEC24B, and this interaction could be promoted or inhibited by the overexpression or downregulation of circSEC24B, respectively (Fig. 4F). On the contrary, RIP assay suggested the endogenous enrichment of circSEC24B by SRPX2 antibody in HCT116 cells, which could be enhanced by upregulation of circSEC24B but not by liner SEC24B (Fig. 4G). The result of RNA-FISH as well as immunofluorescence assays confirmed the mainly cytoplasmic colocalization of circSEC24B and SRPX2 in LoVo and HCT116 cell lines (Fig. 4H, I), indicating that circSEC24B directly regulated and interacted with SRPX2.

### CircSEC24B increased CRC proliferative ability by regulating the protein stability of SRPX2

**Fig. 5:**
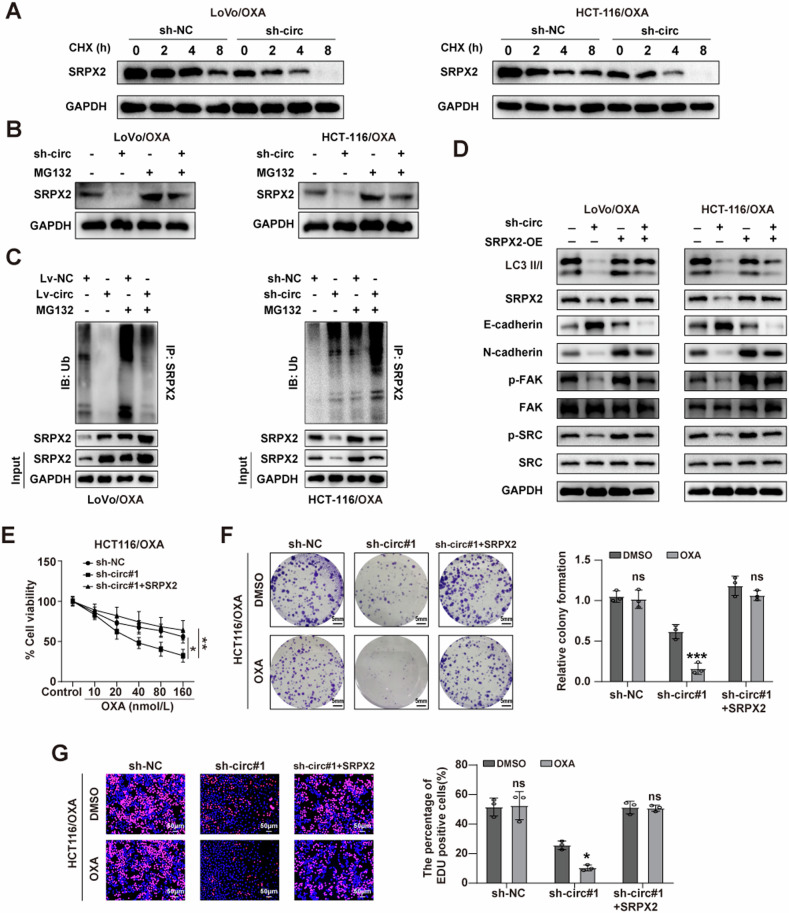
CircSEC24B increased CRC proliferative ability by regulating the protein stability of SRPX2. **A** Western blot assay for SRPX2 with or without knockdown of circSEC24B in the LoVo/OXA and HCT116/OXA cell lines treated with CHX at different time point. **B** The effect of MG132 on SRPX2 protein with or without circSEC24B knockdown in LoVo/OXA and HCT116/OXA cells by western blot assay. **C** The effect of circSEC24B on SRPX2 ubiquitination was evaluated in LoVo/OXA and HCT116/OXA cells treated with or without MG132. **D** The influence of knockdown of circSEC24B and overexpression of SRPX2 on SRPX2, N-cadherin, p-FAK, p-SRC, and E-cadherin were evaluated. The effect of circSEC24B knockdown and/or SRPX2 overexpression on cell viability and proliferation ability were assessed by CCK-8 (**E**), colony formation (**F**), and EDU assays (**G**) in the HCT116/OXA cell line.

Since circSEC24B did not affect the mRNA of SRPX2, but had a critical effect on the protein level of SRPX2. We speculated that circSEC24B increased protein level of SRPX2 by affecting the degradation of SRPX2. To assess whether circSEC24B regulated the SRPX2 protein stability, we measured the protein level of SRPX2 in the LoVo and HCT116 cell lines treated with CHX. Knockdown of circSEC24B in both CRC cells can obviously shorten the half-life of SRPX2 (Fig. 5A). Then we treated the both CRC cells with inhibitor of ubiquitin-proteasome pathway, MG132. The result showed that MG132 could reverse the attenuated protein level of SRPX2 by circSEC24B knockdown (Fig. 5B). In addition, we found that circSEC24B silence facilitated SRPX2 ubiquitination with or without MG132 treatment in CRC cells (Fig. 5C). The collective result indicated that circSEC24B stabilized the SRPX2 protein via suppressing the ubiquitination of SRPX2. Previous studies have shown that SRPX2 promoted cancer progression via activating FAK/SRC/ERK pathway [33, 34]. Next, we further explored the expression of SRPX2 and its downstream proteins with circSEC24B knockdown. In the presence or absence of SRPX2, circSEC24B knockdown inhibited the protein levels of N-cadherin, p-FAK, and p-SRC, promoted the protein levels of E-cadherin in both CRC cells (Fig. 5D). Interestingly, circSEC24B overexpression enables CRC cells to escape anoikis, a programmed cell death triggered by detachment from the matrix. In addition, 3-MA can reverse anoikis resistance induced by circSEC24B overexpression (Fig. S3K, L), suggesting that circSEC24B can influence “Anoikis resistance” by regulating autophagy in CRC-resistant cells. Phenotypically, rescue assays also showed that circSEC24B knockdown blocked the proliferative ability of LoVo cells, which can be reversed by SRPX2 overexpression (Figs. 5E–G and S4A–C). Meanwhile, the cancer-promoting effect of SRPX2 can be reversed by 3-MA (Fig. S4D–K). Collectively, circSEC24B promoted CRC proliferative ability by regulating the protein stability of SRPX2.

### CircSEC24B interplayed with OTUB1 in CRC cells

**Fig. 6:**
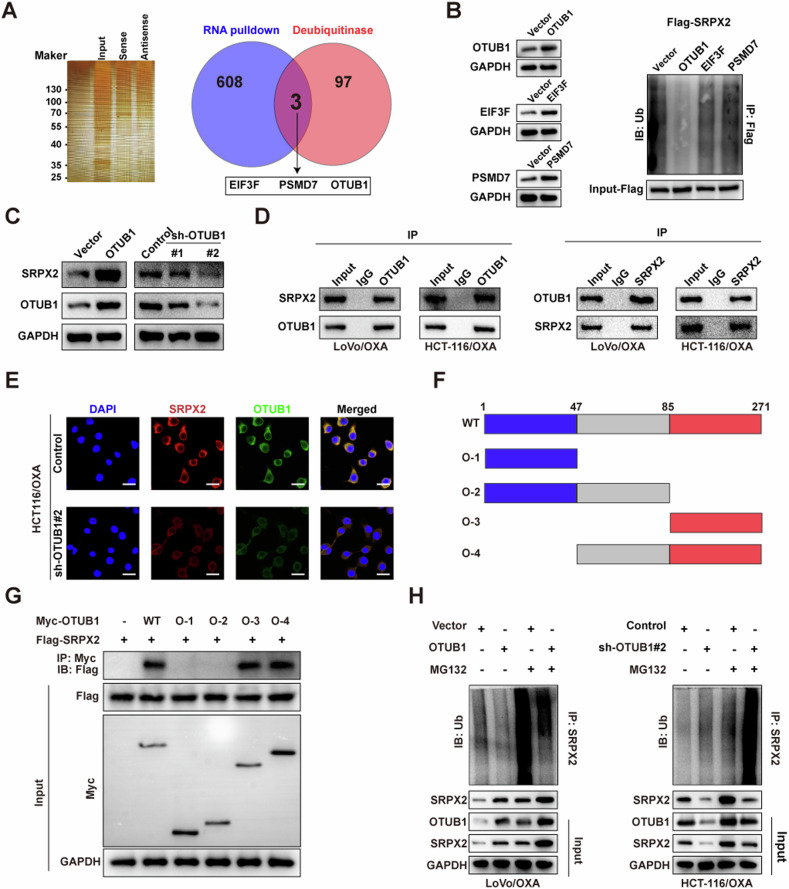
CircSEC24B interplayed with OTUB1 in CRC cells. **A** Biotin-labeled RNA pull-down assay to explore the biological partners of circSEC24B (left). The identified 611 differential expression proteins were used to intersect with the known deubiquitinating enzymes and 3 deubiquitinases were determined (right). **B** The effect of the three deubiquitinases overexpression on the ubiquitination of SRPX2 in the HCT116/OXA cells. **C** The effect of OTUB1 on SRPX2 protein expression level by western blot assay in the HCT116/OXA cells. **D** The mutual co-immunoprecipitation assay to assess the interplay between SRPX2 and OTUB1 in the LoVo/OXA and HCT116/OXA cells. **E** Immunofluorescence assay to assess the location of SRPX2 and OTUB1 in the HCT116/OXA cells transfected with or without sh-OTUB1. **F** Schematic diagram suggesting the domains of OTUB1 truncations. **G** In vitro binding assay indicating the enrichment degree of circSEC24B evaluated through RT-PCR assay (lower panel) following incubation with full-length or truncations versions of Flag-tagged or Myc-tagged recombinant OTUB1 protein (upper panel). **H** The effect of OTUB1 on the ubiquitination level of SRPX2 with or without proteasome inhibitor MG132 in LoVo/OXA and HCT116/OXA cells.

CircRNA is a non-coding RNA and was unlikely to exert a deubiquitinating effect. We guessed that some deubiquitinating enzymes may mediate the deubiquitination of SRPX2. To analyze the possible mechanism underlying the biological impacts of circSEC24B, biotin-labeled RNA pull-down assay were performed to explore the biological partners of circSEC24B. The results of MS suggested that 611 differentially expressed proteins were obtained between the antisense and sense groups. These proteins were intersected with the known deubiquitinating enzymes, and three ubiquitinated proteins were found to potentially regulate SRPX2. (Fig. 6A). Then, the above three genes were overexpressed, and the result showed that only OTUB1 overexpression significantly reduced the ubiquitination of SRPX2 (Fig. 6B). We also found that OTUB1 overexpression increased SRPX2 protein expression level, while OTUB1 knockdown decreased it in both LoVo and HCT116 cell lines (Fig. 6C). In addition, the mutual co-immunoprecipitation assay indicated that SRPX2 and OTUB1 bound to each other in the LoVo and HCT116 cell lines (Fig. 6D). Next, immunofluorescence assay was performed to assess the location of SRPX2 and OTUB1 and the result showed that the two molecules were mainly colocalized in cytoplasm in both LoVo and HCT116 cell lines and knockdown of OTUB1 decrease the cytoplasm presence of SRPX2 (Figs. 6E and S5C). In vitro binding assays revealed that O-3 domain (85-271 amino acids) of the MYC-tagged OTUB1 protein binds to SRPX2 (Fig. 6F, G). Catalytic activity assay of OTUB1 showed that C91S (dead mutant form) is critical for modulation of SRPX2 stability, while OTUB1 ASA (Y26A/D27A/E28A) acts the similar effect to OTUB1 wild type (Fig. S5A,B). Meanwhile, following treated with or without MG132, we found that more SRPX2 was pulled down and decreased ubiquitination level of SRPX2 was observed with OTUB1 overexpression in both CRC cells (Fig. 6H). Overall, these results demonstrate that OTUB1 interacts with and directly deubiquitinates SRPX2.

### CircSEC24B served as a scaffold to enhance the binding of SRPX2 proteins with OTUB1

**Fig. 7:**
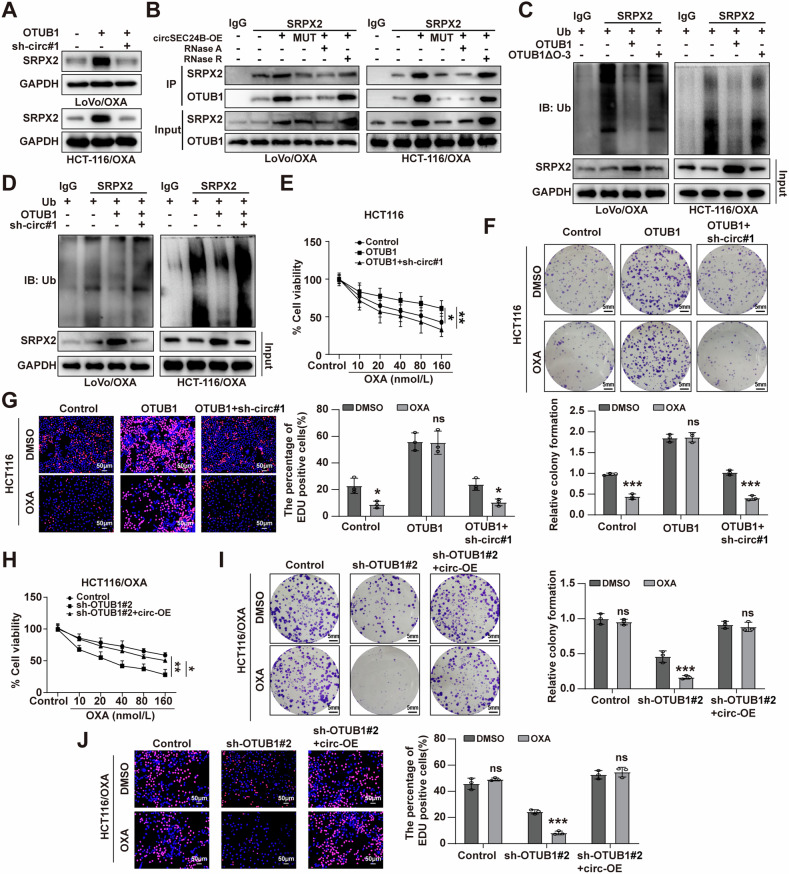
CircSEC24B served as a scaffold to enhance the binding of OTUB1 proteins with SRPX2. **A** The effect of circSEC24B and OTUB1 on the protein level of SRPX2 by western blot assay in the LoVo/OXA and HCT116/OXA cells. **B** Co-IP assay to assess the correlation between SRPX2 and OTUB1 in circSEC24B-WT or circSEC24B-MUT transfected LoVo/OXA and HCT116/OXA cells treated with or without RNAse A or RNAse R. **C** The effect of OTUB1 and its truncation form OTUB1∆O-3 on the ubiquitination level of SRPX2. **D** The effect of OTUB1 and/or circSEC24B knockdown on the ubiquitination level of SRPX2 The effect of OTUB1 overexpression and/or circSEC24B knockdown on cell viability and proliferation ability were evaluated by CCK-8 (**E**), colony formation (**F**) and EDU assays (**G**) in the HCT116 cells treat with OXA or DMSO. The effect of OTUB1 knockdown and/or circSEC24B overexpression on cell viability and proliferation ability was evaluated via CCK-8 (**H**), colony formation (**I**), and EDU assays (**J**) in the HCT116/OXA cells treated with OXA or PBS.

Considering that both circSEC24B and OTUB1 can regulate the expression of SRPX2, and circSEC24B and OTUB1 bind to each other, we hypothesized that circSEC24B functions as a scaffold to enhance the binding of SRPX2 with OTUB1. As expected, western blot results revealed that knockdown of circSEC24B could reverse OTUB1-induced SRPX2 elevation (Fig. 7A). Besides, Co-IP assay showed that the interaction between SRPX2 and OTUB1 was strengthened with circSEC24B transfection, but not circSEC24B-MUT transfection (Fig. 7B). Treatment with RNase A (a RNA endonuclease), but not RNase R (a RNA exonuclease), obviously impaired these interactions due to the resistance of circSEC24B to RNA exonuclease (Fig. 7B). In addition, OTUB1 overexpression decreased the ubiquitination levels of SRPX2, but this effect was attenuated by deletion of O-3 domain of OTUB1 or knockdown of circSEC24B (Fig. 7C, D). To further explore whether OTUB1 enhanced the progression of CRC cells in a circSEC24B-dependent manner, OTUB1-overexpression vector or/and sh-circSEC24B vector was utilized. The results exhibited that OTUB1 significantly elevated CRC cell viability and proliferation ability, which could be reversed by circSEC24B knockdown (Figs. 7E–G and S5D,F). Meanwhile, the cancer-inhibiting role of OTUB1 knockdown could be reversed by overexpression of circSEC24B (Figs. 7H–J and S5G, I). Collectively, the above results showed that OTUB1 inhibited ubiquitination and degradation of SRPX2 in a circSEC24B-dependent manner.

### OTUB1 regulated SRPX2 in an acetylation-dependent approach

**Fig. 8:**
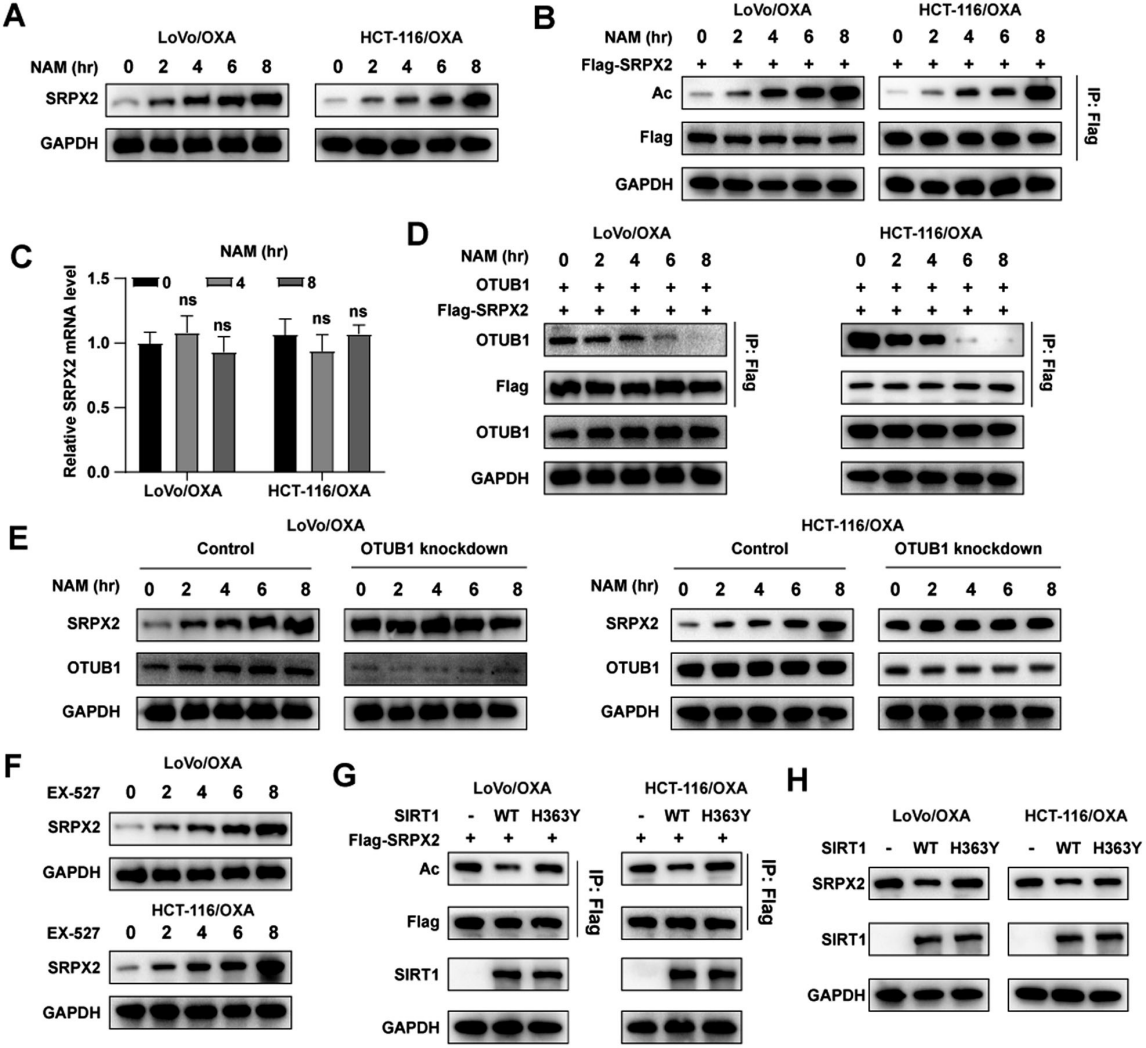
OTUB1 regulates SRPX2 in an acetylation-dependent approach. **A** Western blot assay for SRPX2 protein in LoVo/OXA and HCT116/OXA cells with NAM at different points. **B** Western blot assay for SRPX2 acetylation levels in LoVo/OXA and HCT116/OXA cells with NAM at different points. The LoVo/OXA and HCT116/OXA cells were transfected with SRPX2-FLAG. **C** RT-qPCR assay for SRPX2 mRNA levels in LoVo/OXA and HCT116/OXA cells treated with NAM at different points. **D** Co-IP assay was used to evaluated the effect of NAM on binding degree of SRPX2 to OTUB1. **E** Western blot assay was applied to evaluate whether NAM regulation of SRPX2 expression depends on the normal expression of OTUB1. **F** Western blot assay for SRPX2 protein level in HCT116/OXA cells treated with EX-527 at different points. **G** Co-IP assay was used to evaluated the effect of WT-SIRT1 or Mut-SIRT1 on SRPX2 acetylation levels. **H** Western blot assay was used to evaluated the effect of WT-SIRT1 or Mut-SIRT1 on SRPX2 protein levels.

Various studies suggested that protein stability was also regulated by acetylation. For example, Shimizu et al. found the interaction between protein acetylation and ubiquitination can control MCL1 protein stability [35]. Wang et al. revealed that E3 ligase RNF5 promoted the expression of PHGDH in an acetylation-dependent manner [36]. To further explore whether the interaction between OTUB1 and SRPX2 was affected by the acetylation level of SRPX2. The CRC cells were treated with 5 mM NAM and the result of the western blot assay showed that SRPX2 protein as well as its acetylation level increased with NAM treatment in a dose-dependent approach (Fig. 8A, B).

Following this, RT-qPCR assay was performed to assess the mRNA level of SRPX2 after NAM treatment. The result indicated that NAM did not affect the mRNA of SRPX2, suggesting that the upregulated SRPX2 was not caused by mRNA upregulation (Fig. 8C). Studies showed that acetylation had an important effect on regulation of protein degradation [37, 38]. Given that SRPX2 is degraded by the ubiquitin-protease system and OTUB1 acts as its deubiquitinating enzyme, it is reasonable to assume that OTUB1 was closely associated with the accumulation of SRPX2 protein in an acetylation-dependent manner. As expected, the co-IP experiment results indicated that the binding of OTUB1 to SRPX2 was weakened in a dose-dependent manner after the treatment of NAM. In addition, western blot assay indicated that NAM elevated SRPX2 expression depending on normal OTUB1 expression (Fig. 8E). These results manifested that OTUB1 deubiquitinated SRPX2 in an acetylation-dependent manner.

Previous studies have shown that SIRT1 played a critical role in protein stability [39, 40]. To investigated whether SIRT1 mediated the acetylation-induced elevation of SRPX2, we treated the CRC cells with EX-527, an inhibitor of SIRT1. We found that EX-527 treatment, like NAM, also upregulated the expression of SRPX2 (Fig. 8F). Moreover, we explored whether SIRT1 had a critical effect on SRPX2 expression as well as its acetylation level via ectopically SIRT1 overexpression and its deacetylase-defective mutant (H363Y) in CRC cells. The result showed that the overexpression of SIRT1 led to a lower acetylation level of SRPX2, whereas no change was observed in the mutant (H363Y) group (Fig. 8G). Consistently, decreased SRPX2 protein levels were also found in SIRT1 overexpression group, but not in the mutant (H363Y) group (Fig. 8H). These results suggested that OTUB1 regulated SRPX2 in an acetylation-dependent approach.

## Discussion

**Fig. 9:**
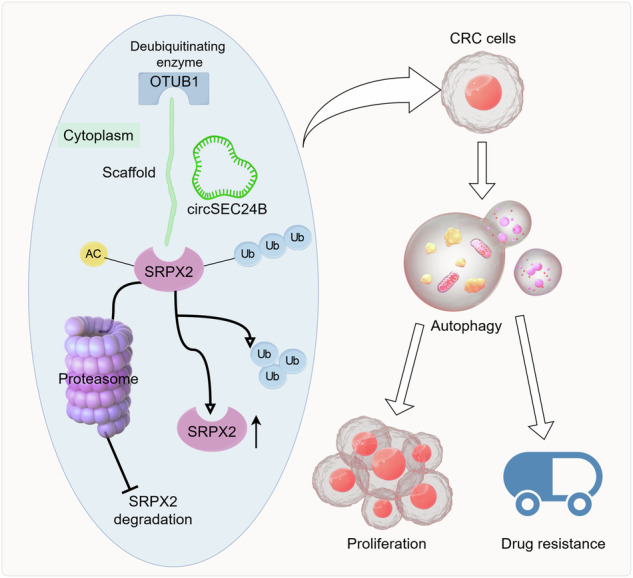
Mechanism diagram. Schematic illustration of CircSEC24B/OTUB1/SRPX2 signaling contributes to chemoresistance of CRC.

Our study provides compelling evidence for the critical role of circSEC24B in CRC progression, particularly in the context of chemoresistance to OXA. The upregulation of circSEC24B in CRC cell lines and tissues compared to normal counterparts underscores its potential as a biomarker for early CRC detection and as a therapeutic target for CRC treatment. The discovery that circSEC24B promotes CRC cell proliferation and drug resistance through modulation of autophagy represents a significant advance in our understanding of CRC pathogenesis. Autophagy is a cellular process that plays a dual role in cancer, acting as a tumor suppressor in early stages but promoting progression and drug resistance in later stages. Our findings suggest that circSEC24B may contribute to the switch from autophagy-mediated tumor suppression to autophagy-mediated drug resistance in CRC. Furthermore, our identification of SRPX2 as a downstream target of circSEC24B sheds light on the molecular mechanisms by which circRNAs regulate cancer progression. By stabilizing SRPX2 protein through suppression of its ubiquitination, circSEC24B activates oncogenic signaling pathways, such as FAK/SRC/ERK, that promote CRC cell proliferation, migration, and invasion. This novel mechanism highlights the intricate interplay between circRNAs, ubiquitination, and protein stability in cancer biology. Additionally, our findings regarding the interaction between circSEC24B and OTUB1, a deubiquitinating enzyme, reveal a previously unrecognized layer of regulation in CRC. The ability of OTUB1 to deubiquitinate SRPX2 in an acetylation-dependent manner suggests that protein acetylation may play a critical role in modulating the effects of circRNAs and deubiquitinating enzymes on cancer progression (Fig. 9).

While our results are promising, several limitations should be acknowledged. Firstly, our findings are based primarily on in vitro and in vivo models, and further validation in human CRC samples is necessary to confirm their clinical relevance. This includes assessing the expression levels of circSEC24B and SRPX2 in CRC tissues and correlating them with patient outcomes. Secondly, the precise molecular mechanisms by which circSEC24B regulates autophagy and protein stability remain to be fully elucidated. Future studies should aim to identify additional interacting partners of circSEC24B and SRPX2, as well as downstream signaling pathways that are activated by their interaction. This will provide a more comprehensive understanding of the role of circSEC24B in CRC progression. Thirdly, our study focused on the effects of circSEC24B on OXA resistance, but the role of circSEC24B in resistance to other chemotherapeutics remains to be investigated. It is possible that circSEC24B may confer resistance to a broader range of chemotherapeutics through similar mechanisms, and further studies are needed to address this question.

Given the emerging importance of circRNAs in cancer biology, it is likely that circSEC24B or similar circRNAs may play critical roles in other tumor types as well. The mechanisms identified in our study, including modulation of autophagy, protein stability, and deubiquitination, are common themes in cancer progression across different tumor types. Therefore, future studies investigating the expression and function of circSEC24B in other tumors may reveal novel insights into cancer biology and identify new therapeutic targets. For instance, Peng et al. indicated that circCUL2 regulated gastric cancer malignant transformation and cisplatin resistance via regulating autophagy activation by miR-142-3p/ROCK2 [19]. Gao et al. suggested circPARD3 drove malignant progression and chemoresistance of laryngeal squamous cell carcinoma via suppressing autophagy by the PRKCI-Akt-mTOR pathway (Gao et al. [41]). Yu et al. found that circCEMIP promotes anoikis resistance by enhancing protective autophagy in prostate cancer cells (Yu et al. [42]). It is possible that circSEC24B or related circRNAs may contribute to the progression of these tumors through similar mechanisms as those identified in our study.

Future studies should aim to validate the clinical relevance of circSEC24B in CRC and other tumor types by assessing its expression levels in patient samples and correlating them with disease outcomes. In addition, functional studies are needed to further elucidate the molecular mechanisms by which circSEC24B regulates autophagy, protein stability, and cancer progression. These studies may identify novel therapeutic targets for CRC and other cancers, as well as inform the development of more effective chemotherapeutics that can overcome drug resistance.

## Supplementary information

Original Data

Supplementary file

Raw_PCR

## Data Availability

The datasets used and/or analyzed during the current study are available from the corresponding author upon reasonable request.
