# Supplementary material for: CircSEC24B activates autophagy and induces chemoresistance of colorectal cancer via OTUB1-mediated deubiquitination of SRPX2
Source: Cell Death Dis. 2024 Sep 27;15(9):693. doi: 10.1038/s41419-024-07057-y (PMC11436887; doi:10.1038/s41419-024-07057-y)

Raw_Fig 3

Fig 3

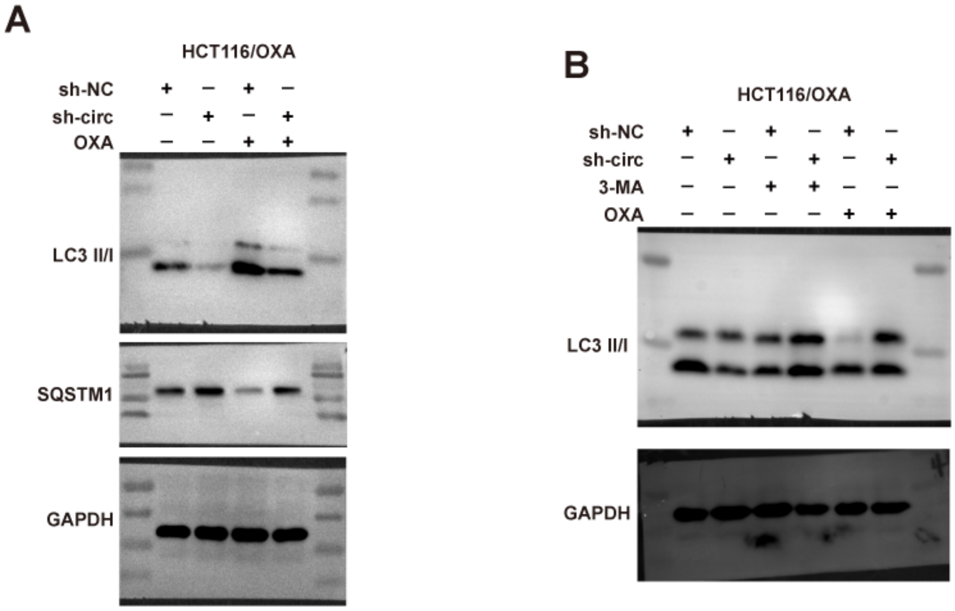

Raw_Fig 4

Fig 4

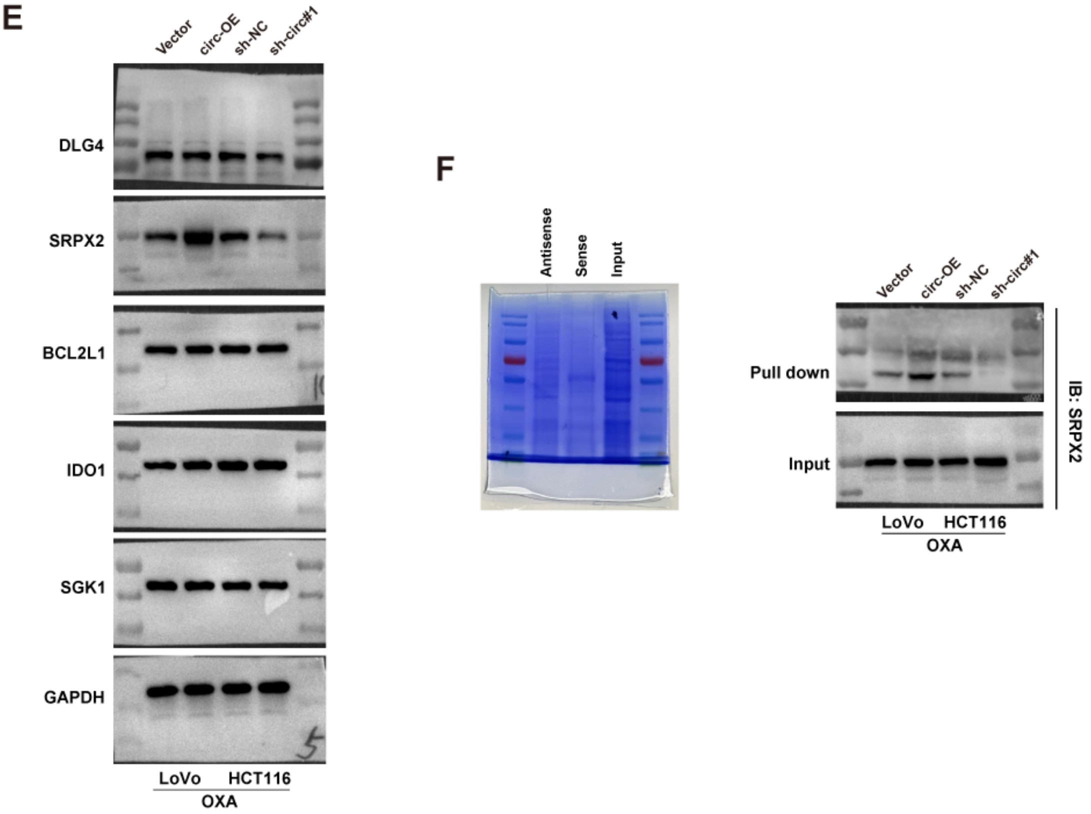

Raw_Fig 5

Fig 5

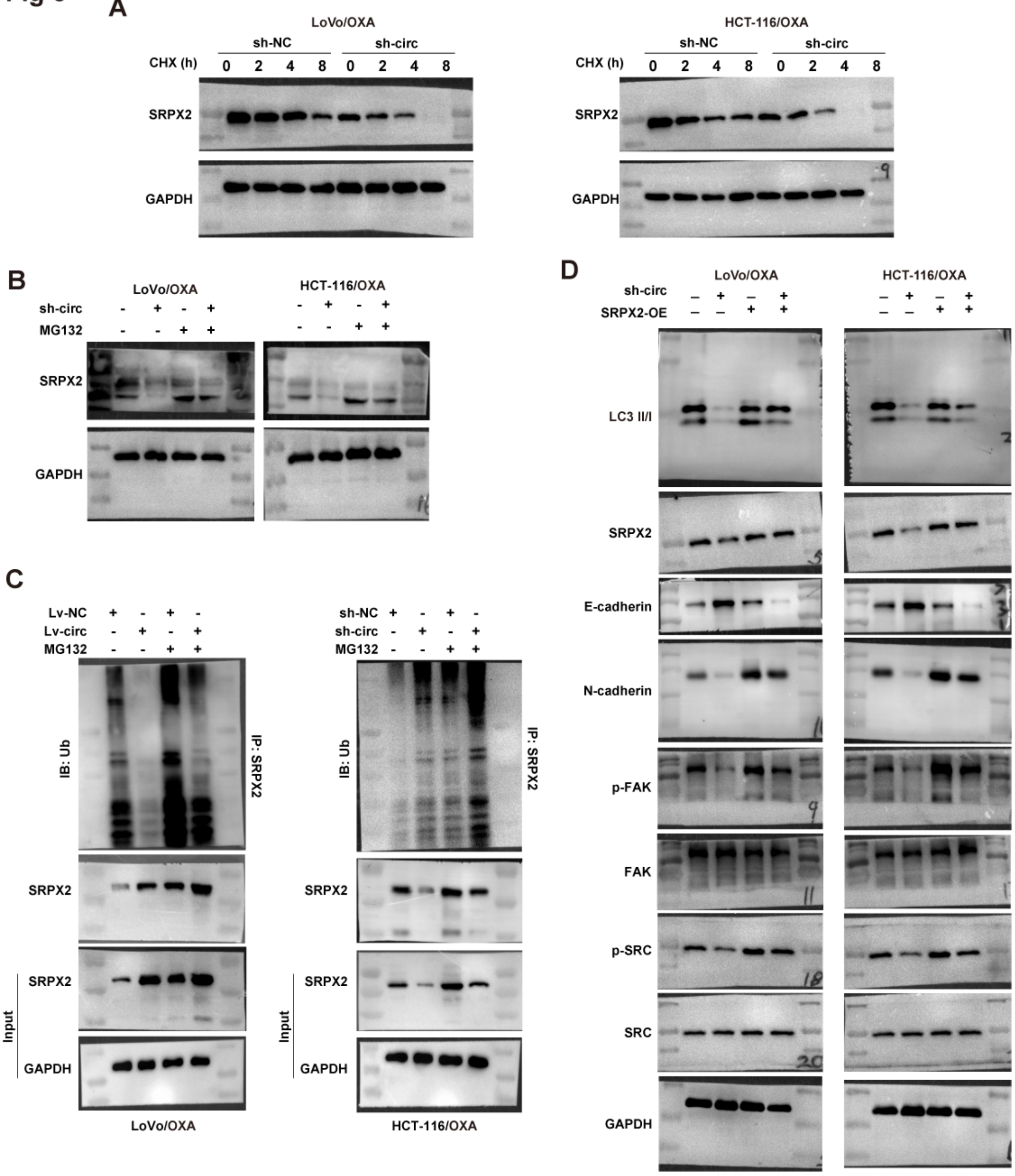

Raw_Fig 6

Fig 6

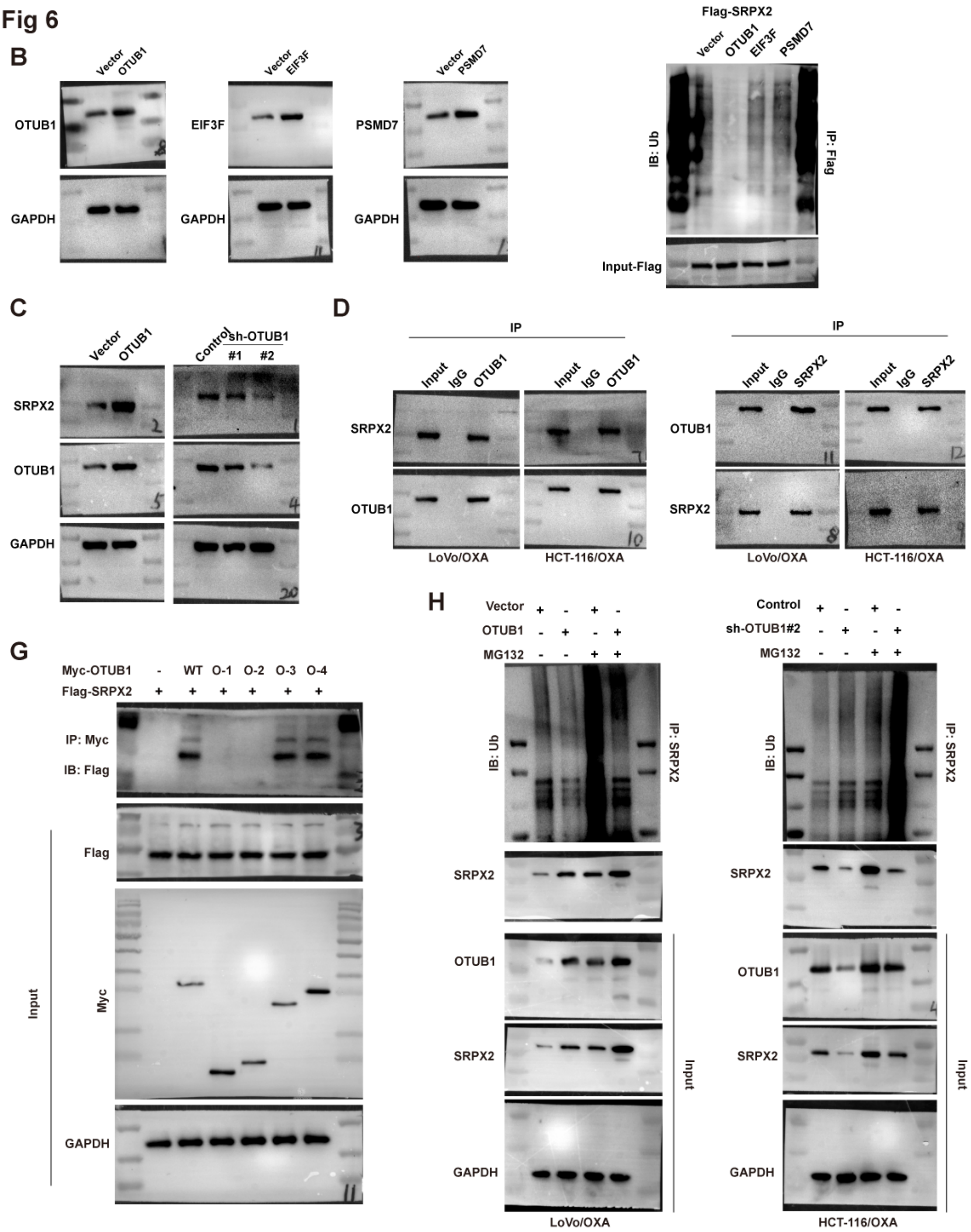

Raw_Fig 7

Fig 7

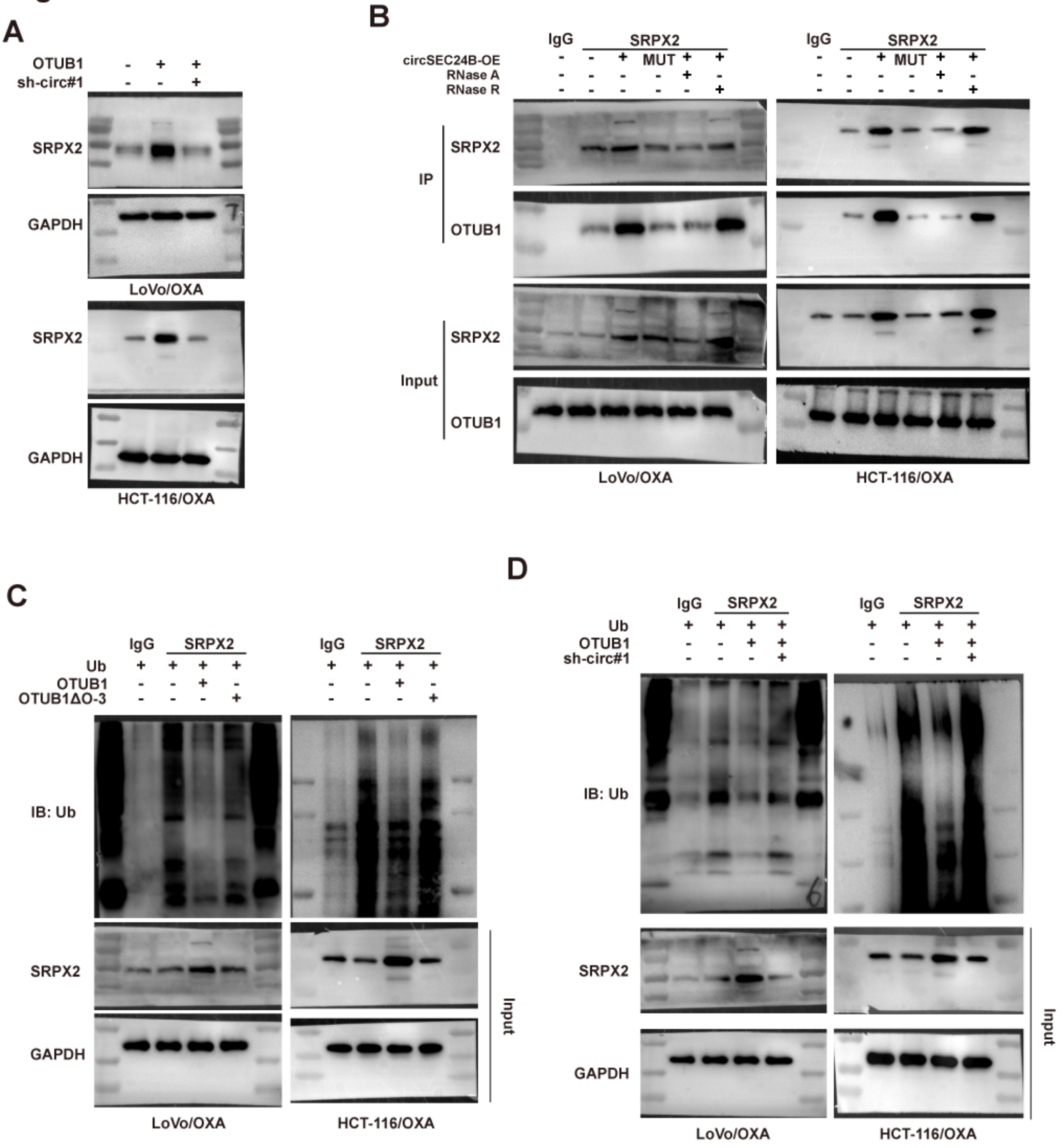

Raw_Fig 8

Fig 8

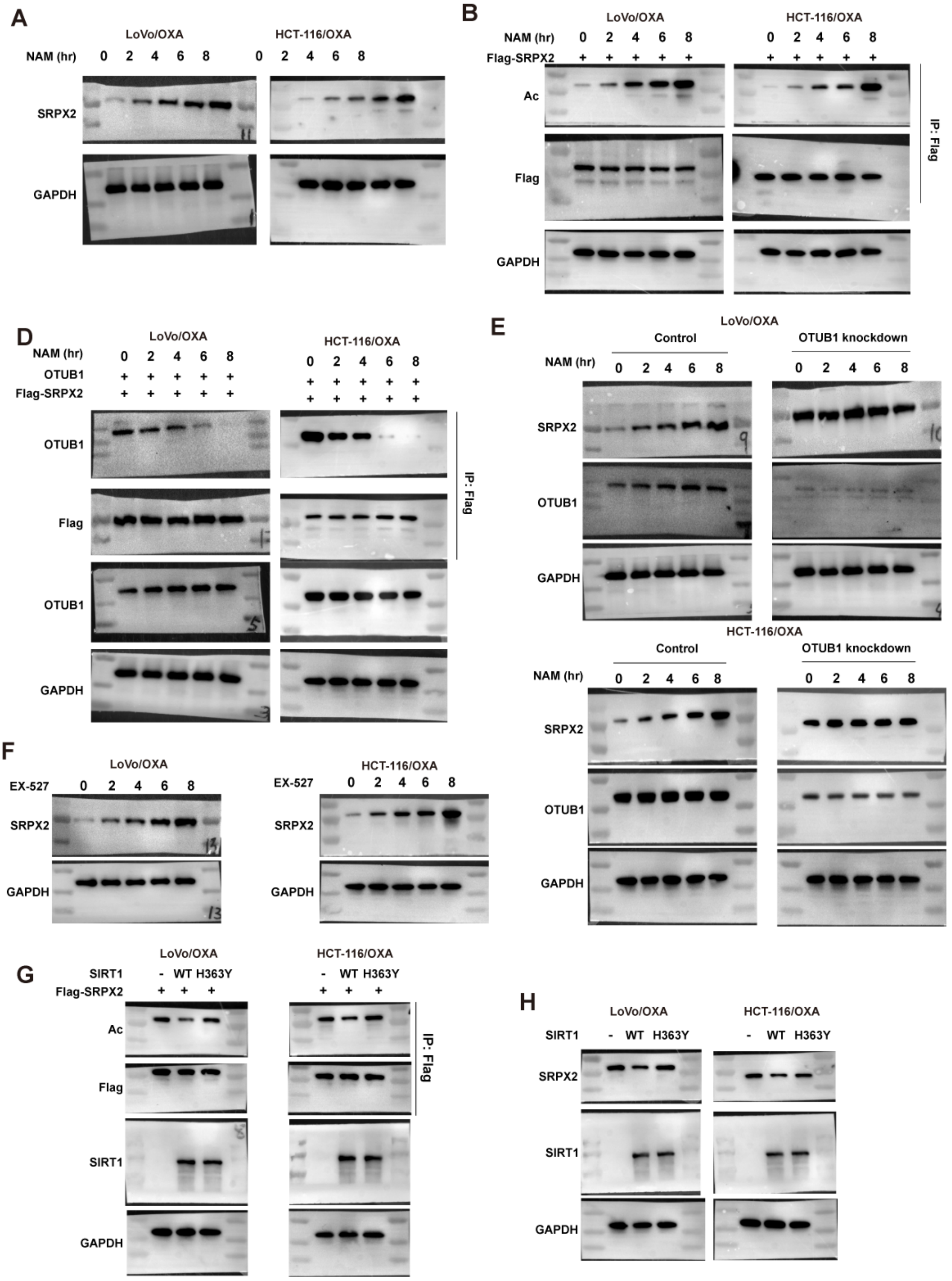

Raw_Fig S3

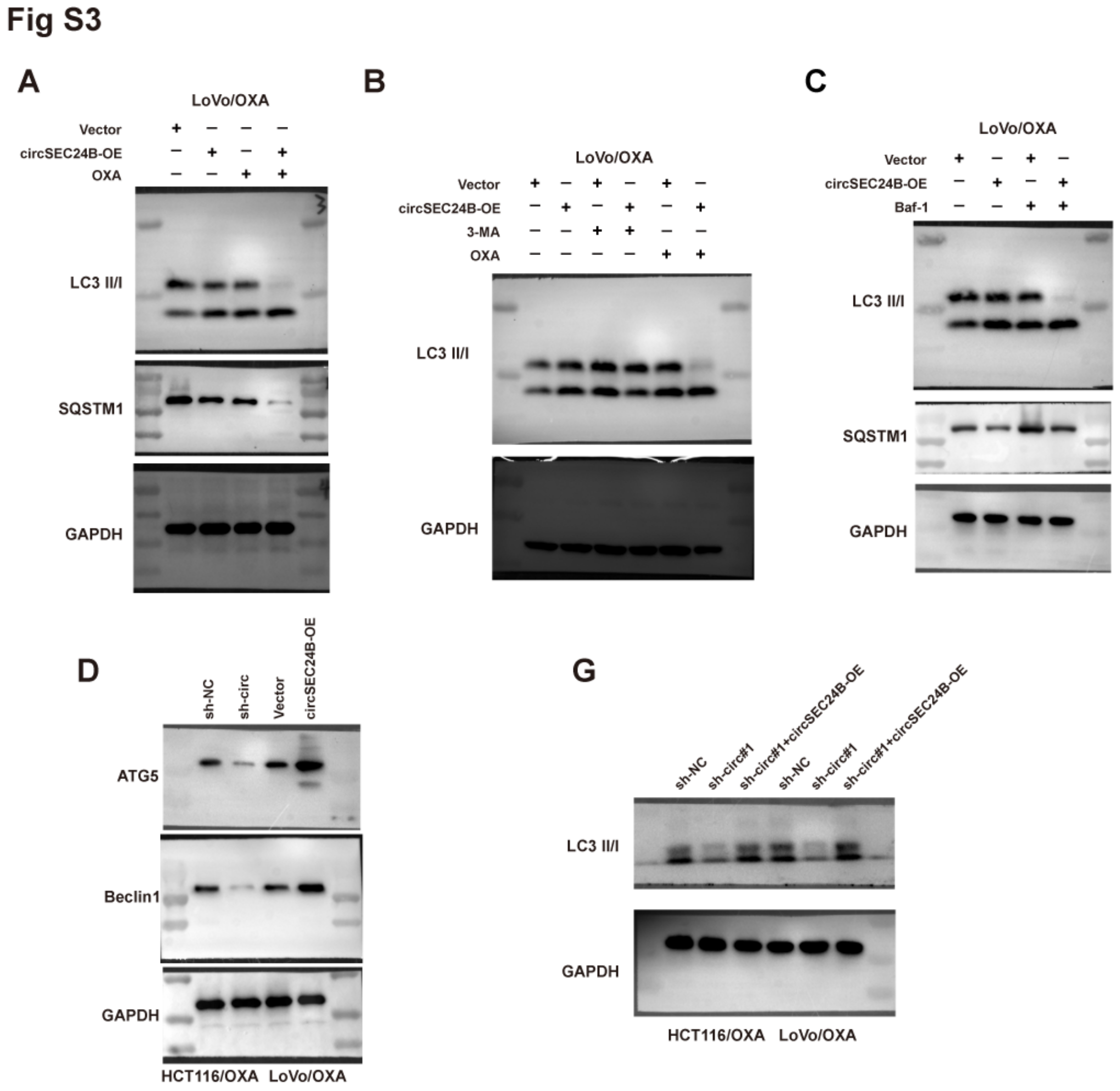
Fig S3
A
LoVo/OXA
Vector
circSEC24B-OE
OXA
LC3 II/I
SQSTM1
GAPDH
B
LoVo/OXA
Vector
circSEC24B-OE
3-MA
OXA
LC3 II/I
GAPDH
C
LoVo/OXA
Vector
circSEC24B-OE
Baf-1
LC3 II/I
SQSTM1
GAPDH
D
sh-NC
sh-circ
Vector
circSEC24B-OE
ATG5
Beclin1
GAPDH
HCT116/OXA
LoVo/OXA
G
sh-NC
sh-circ#1
sh-circ#1+circSEC24B-OE
sh-NC
sh-circ#1
sh-circ#1+circSEC24B-OE
LC3 II/I
GAPDH
HCT116/OXA
LoVo/OXA

Raw_Fig S5

Fig S5

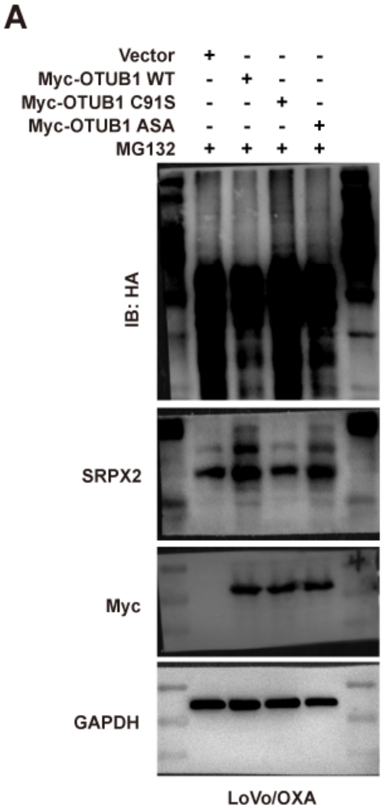

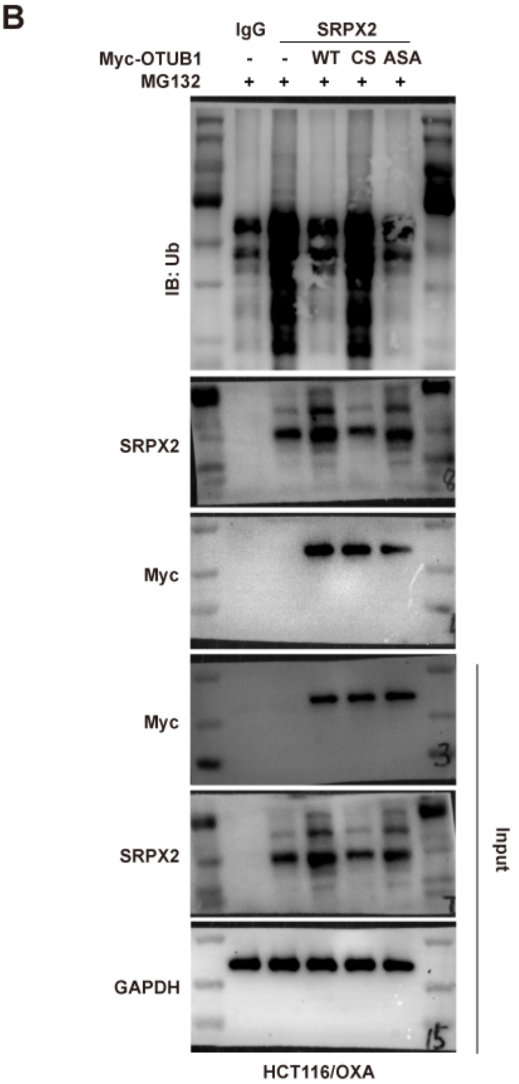

Supplement: Supplementary file 1 — Original Data [file 41419_2024_7057_MOESM1_ESM.pdf]
