# Supplementary material for: CircSEC24B activates autophagy and induces chemoresistance of colorectal cancer via OTUB1-mediated deubiquitination of SRPX2
Source: Cell Death Dis. 2024 Sep 27;15(9):693. doi: 10.1038/s41419-024-07057-y (PMC11436887; doi:10.1038/s41419-024-07057-y)
Supplement: Supplementary file 2 — Supplementary file [file 41419_2024_7057_MOESM2_ESM.pdf]

Table S1. Detailed clinical information of enrolled samples.

ID	Size	2cm?	TNM	I-IV	Age	Location	Sex	Vascular	Nerve	Lymph_Node
3433008	2cm*1.8cm	⩾2cm	pT2N0M0	1	80	Rectum	Male	No	No	0
2090006	1.5cm*2cm	⩾2cm	pT3N2M0	3	44	Rectum	Male	Yes	Yes	3
3397301	2cm*1.8cm	⩾2cm	pT1N0M0	1	59	Rectum	Male	No	No	0
3383935	1.5cm*1.1cm*0.6cm	<2cm	pT4N1M0	3	67	Rectum	Female	No	No	2
3316138	1.5cm*1.3cm	<2cm	pT3N2M0	3	49	Rectum	Male	No	No	3
3161873	1.5cm*1.3cm	<2cm	pT1N0M0	1	72	Colon	Male	No	No	0
3359158	2cm*1cm*0.5cm	⩾2cm	pT1N1M0	3	50	Rectum	Male	Yes	Yes	1
3393435	1.5cm*2cm	⩾2cm	pT1N1M0	3	69	Rectum	Female	No	No	1
3431903	1.5cm*2cm	⩾2cm	pT1N1M0	3	56	Rectum	Female	Yes	Yes	1
2180896	1.8cm*1.2cm*0.5cm	<2cm	pT3N1M0	3	54	Rectum	Male	No	Yes	3
3434917	2cm*1.7cm	⩾2cm	pT3N3M0	3	61	Rectum	Male	Yes	Yes	6
3377910	1.9cm*2cm	⩾2cm	pT3N0M0	2	55	Rectum	Female	No	Yes	0
3273310	2cm*1.8cm	⩾2cm	pT2N1M0	3	63	Rectum	Female	Yes	Yes	1
3010151	1.4cm*0.9cm*0.6cm	<2cm	pT4N1M0	3	61	Rectum	Male	Yes	Yes	1
1844572	1.2cm*0.1cm	<2cm	pT2N0M0	1	71	Rectum	Male	No	No	0
3397111	2cm*1cm*0.5cm	⩾2cm	pT4N1M0	3	64	Colon	Female	No	Yes	1
2103225	1.8cm*1.2cm*0.5cm	<2cm	pT3N1M0	3	64	Rectum	Male	No	No	1
3388156	2cm*1.5cm	⩾2cm	cT3N2M1	4	41	Rectum	Female	Yes	Yes	4
3367922	0.2cm*0.2cm	<2cm	pT3N1M0	3	52	Rectum	Female	No	No	1
8149263	2cm*1.8cm	⩾2cm	pT4N2M0	3	70	Colon	Male	Yes	Yes	3
3392459	0.2cm*0.2cm	<2cm	pT3N0M0	2	72	Rectum	Male	No	No	0
3306678	1.5cm*0.1cm	<2cm	pT3N0M0	2	66	Rectum	Female	No	No	0
3330952	0.7cm*0.5cm	<2cm	pT1N1M0	3	33	Colon	Male	Yes	Yes	1

3330952	1.9cm*2cm	⩾2cm	pT4N1M0	3	58	Rectum	Male	Yes	Yes	1
3338759	0.2cm*0.2cm	<2cm	pT1N1M0	3	53	Colon	Male	Yes	Yes	1
3272936	1.5cm*1.3cm*0.5cm	<2cm	pT1N1M0	3	53	Colon	Male	No	No	1
3048142	2cm*1.8cm	⩾2cm	pT1N1M0	3	38	Rectum	Female	Yes	Yes	1
3294232	1.9cm*2cm	⩾2cm	pT3N1M1	4	76	Rectum	Male	Yes	Yes	2
3275164	1.5cm*0.1cm	<2cm	pT3N1M1	4	63	Colon	Male	Yes	Yes	2
3229502	0.2cm*0.2cm	<2cm	cT3N1M1	4	58	Rectum	Male	Yes	Yes	1
3149132	0.7cm*0.5cm	<2cm	cT2N0M1	4	35	Rectum	Male	Yes	Yes	0
3372759	1.9cm*2cm	⩾2cm	pT3N1M1	4	56	Colon	Male	Yes	Yes	1
3369561	2cm*1.5cm	⩾2cm	pT3N2M0	3	68	Colon	Female	Yes	Yes	4
3097861	2cm*1.8cm	⩾2cm	cT3N1M1	4	52	Colon	Male	Yes	Yes	1
3361459	2cm*1.8cm	⩾2cm	pT4N0M0	2	56	Colon	Female	No	Yes	0
3319446	2cm*1.8cm	⩾2cm	pT4N2M1	4	60	Rectum	Male	Yes	Yes	3
3208040	1.5cm*1cm*0.6cm	<2cm	pT3N0M1	4	61	Rectum	Male	Yes	Yes	0
3400880	1.5cm*1.3cm*0.5cm	<2cm	pT3N1M1	4	46	Rectum	Female	Yes	Yes	2
3149132	0.2cm*0.2cm	<2cm	cT2N0M1	4	35	Rectum	Male	Yes	Yes	0
3325247	1.9cm*2cm	⩾2cm	pT3N2M0	3	47	Rectum	Female	Yes	Yes	3
3393113	2cm*1.5cm	⩾2cm	cT0N1M1	4	69	Colon	Female	Yes	Yes	1
3384474	0.2cm*0.2cm	<2cm	pT4N2M1	4	64	Rectum	Male	Yes	Yes	3
3182215	1.9cm*2cm	⩾2cm	cT0N0M1	4	64	Colon	Male	Yes	Yes	0
3333325	2cm*1.5cm	⩾2cm	pT1N0M1	4	56	Rectum	Male	Yes	Yes	0
3355625	1.9cm*2cm	⩾2cm	cT4N1M1	4	52	Colon	Female	Yes	Yes	1
3402431	2cm*1.5cm	⩾2cm	pT3N1M0	3	48	Rectum	Male	No	No	2
3275164	1.5cm*0.1cm	<2cm	pT4N1M1	4	63	Colon	Male	Yes	Yes	2
3281256	2cm*1.8cm	⩾2cm	pT3N0M1	4	50	Colon	Male	Yes	Yes	0

3383511	2cm*1.8cm	⩾2cm	pT3N1M0	3	53	Colon	Male	No	No	1
3022077	0.2cm*0.2cm	<2cm	pT3N1M1	4	70	Colon	Male	Yes	Yes	1
3207289	0.2cm*0.2cm	<2cm	pT4N1M1	4	56	Colon	Male	Yes	Yes	1
3355291	1.9cm*2cm	⩾2cm	cT3N1M1	4	52	Rectum	Male	Yes	Yes	1
3377131	1.5cm*1.3cm*0.5cm	<2cm	pT3N1M0	3	71	Colon	Female	No	No	2
3369561	1.2cm*0.1cm	<2cm	pT3N2M1	4	53	Colon	Female	Yes	Yes	3
3286543	0.7cm*0.5cm	<2cm	pT4N1M1	4	69	Rectum	Female	Yes	Yes	1
3333325	0.7cm*0.5cm	<2cm	pT1N0M1	4	56	Rectum	Male	Yes	Yes	0
3413191	2cm*1.8cm	⩾2cm	pT4N2M1	4	50	Colon	Male	Yes	Yes	3
3374878	0.2cm*0.2cm	<2cm	pT4N1M0	3	38	Rectum	Female	No	No	2
3397157	1.5cm*0.1cm	<2cm	pT3N1M0	3	59	Colon	Male	No	No	1
3387473	1.5cm*1cm*0.8cm	<2cm	pT4N0M0	2	63	Colon	Male	No	No	0
3358414	2cm*1.5cm	⩾2cm	pT4N1M1	4	53	Colon	Female	Yes	Yes	2
3391356	2cm*1.5cm	⩾2cm	PT4N1M0	3	57	Colon	Male	No	No	2
3233069	2cm*1.5cm	⩾2cm	pT3N2M1	4	55	Colon	Female	Yes	Yes	3
3339160	1.9cm*2cm	⩾2cm	PT3N0M0	2	74	Rectum	Male	No	No	0
3220587	2cm*1.5cm	⩾2cm	pT4N2M0	3	41	Colon	Female	No	No	3
3163082	2cm*1.8cm	⩾2cm	pT3N0M1	4	50	Rectum	Female	Yes	Yes	0
3345401	2cm*1.5cm	⩾2cm	pT3N1M1	4	63	Rectum	Male	Yes	Yes	2
3347436	0.7cm*0.5cm	<2cm	cT4N1M1	4	63	Colon	Female	Yes	Yes	2

Table S2. The sequence of PCR primers and Oligonucleotide sets used for short hairpin RNAs, or probe.

Primer	Sequence
hsa_circ_0001436	Forward: 5′- TCGTCCCTCAGCCTTCAAAA-3′
(Divergent)	Reverse: 5′- TCTGGGTAACTGCCTCCTTC-3′
hsa_circ_0001436	Forward: 5'- GCTTCTCCAATGCCCAACAG-3'
(Convergent)	Reverse: 5'- CAGGCTGAAGTGTTGGATAGC-3'
hsa_circ_0089405	Forward: 5′- GGAGAAGAAGCCCGAGACC-3′
(Divergent)	Reverse: 5′- CACAGGAGACAAGGGTGGAG-3′
hsa_circ_0089405	Forward: 5′- GTCCCTCCACCCTTGTCTC-3′
(Convergent)	Reverse: 5′- CTTGCTCTGAGTGGCGGAT-3′
hsa_circ_0050053	Forward: 5′- TTCTTCTAGGTGCAGGGCC-3′
(Divergent)	Reverse: 5′- CTTCTCAAACACCTGCCCAC-3′
hsa_circ_0050053	Forward: 5′- TCCCGCTTCTGGTACTTTGT-3′
(Convergent)	Reverse: 5′- AGCCAGATCCCGAAGTTCTT-3′
hsa_circ_0003270	Forward: 5′- TACTTCTTTGGTGGCTGCAC-3′
	Reverse: 5′- TTTCCTCCCACAGCTCCTTC-3′
hsa_circ_0077321	Forward: 5′- CGTCTTCATGGGATGGATGC-3′
	Reverse: 5′- CTTCTCTAGCAGCCTCCTCC-3′
hsa_circ_0011551	Forward: 5′- CCATGGTTCAAGCTGCTTCA-3′
	Reverse: 5′- ACCTGAGAACCGAACAGTCT-3′
hsa_circ_0042988	Forward: 5′- ACAATACAAGGCAACAAAC -3′
	Reverse: 5′- ATAGGAGCCATCATAACAC -3′
hsa_circ_0067393	Forward: 5′- GACCACCAGAAATCTACAGTGA-3′
	Reverse: 5′- TGTTTTCCTTGTTGTGGTTAACC-3′
hsa_circ_0003597	Forward: 5′- TGAACACCAGAGGCACCTAC-3′
	Reverse: 5′- AACTGTACATCCTGCCAGCC-3′
hsa_circ_0015421	Forward: 5′- TTCACACGTTGGCATGCTAC-3′
	Reverse: 5′- TGTTTCAGCAAAAGAGGGCC-3′
GAPDH	Forward: 5′- AGAAGGCTGGGGCTCATTTG -3′
	Reverse: 5′- AGGGGCCATCCACAGTCTTC -3′
SEC24B	Forward: 5′- CATTAGATTGCTCGGGACA -3′
	Reverse: 5′- CAAAGAAGTTACCGTGAAAA -3′
DLG4	Forward: 5’- TTCAACATCGTGGGTGGCG -3’
	Reverse: 5’- ATGGCTGGCATTTCGGAGG -3’
SRPX2	Forward: 5’- CCACTGTGATGGCGGTTAT -3’
	Reverse: 5’- GATGAGGAGTCGCTGTTTC -3’
BCL2L1	Forward: 5’- CTGAATCGGAGATGGAGACC -3’
	Reverse: 5’- GAGTGAGCCCAGCAGAACC -3’
IDO1	Forward: 5′- TCATTTCGTGATGGAGACT -3′
	Reverse: 5′- TTGCCTTGAATACAGTAGGA -3’
SGK1	Forward: 5′- GGACTGTGGACTGGTGGTG -3′
	Reverse: 5′- AGGCTCTTCGGTAAACTCG -3’
sh-circSEC24B#1	5'CCGGGTATCCTCAAGTGTTGACAGCTCGAGCTGTCAACACTTGAGG ATACTTTTTG -3' (sense);

	5'AATTCAAAAAGTATCCTCAAGTGTTGACAGCTCGAGCTGTCAACACTTGAGGATAC -3' (antisense)
sh-circSEC24B#2	5'CCGGCCTCAAGTGTTGACAGCTCTCTCGAGAGAGCTGTCAACACTTGAGGTTTTTG -3' (sense);
	5'AATTCAAAAACCTCAAGTGTTGACAGCTCTCTCGAGAGAGCTGTCAACACTTGAGG -3' (antisense)
sh-OTUB1#1	5'CCGGGCAGGACCGAATTCAGCAAGACTCGAGTCTTGCTGAATTCGGTCCTGCTTTTTG -3' (sense);
	5'AATTCAAAAAGCAGGACCGAATTCAGCAAGACTCGAGTCTTGCTGAATTCGGTCCTGC -3' (antisense)
sh-OTUB1#2	5'CCGGGCAAGAGATTGCTGTGCAGAACTCGAGTTCTGCACAGCAATCTCTTGCTTTTTG -3' (sense);
	5'AATTCAAAAAGCAAGAGATTGCTGTGCAGAACTCGAGTTCTGCACAGCAATCTCTTGC -3' (antisense)
sh-Scb	5'CCGGGCGAACGATCGAGTAAACGGACTCGAGTCCGTTTACTCGATCGTTCGCTTTTT-3' (sense);
	5'AATTCAAAAAGCGAACGATCGAGTAAACGGACTCGAGTCCGTTTACTCGATCGTTCGC-3' (antisense)
circSEC24B probe	CAGCAGTATCCTCAAGTGTTGACAGCTCTT (antisense)
	AAGAGCTGTCAACACTTGAGGATACTGCTG (sense)

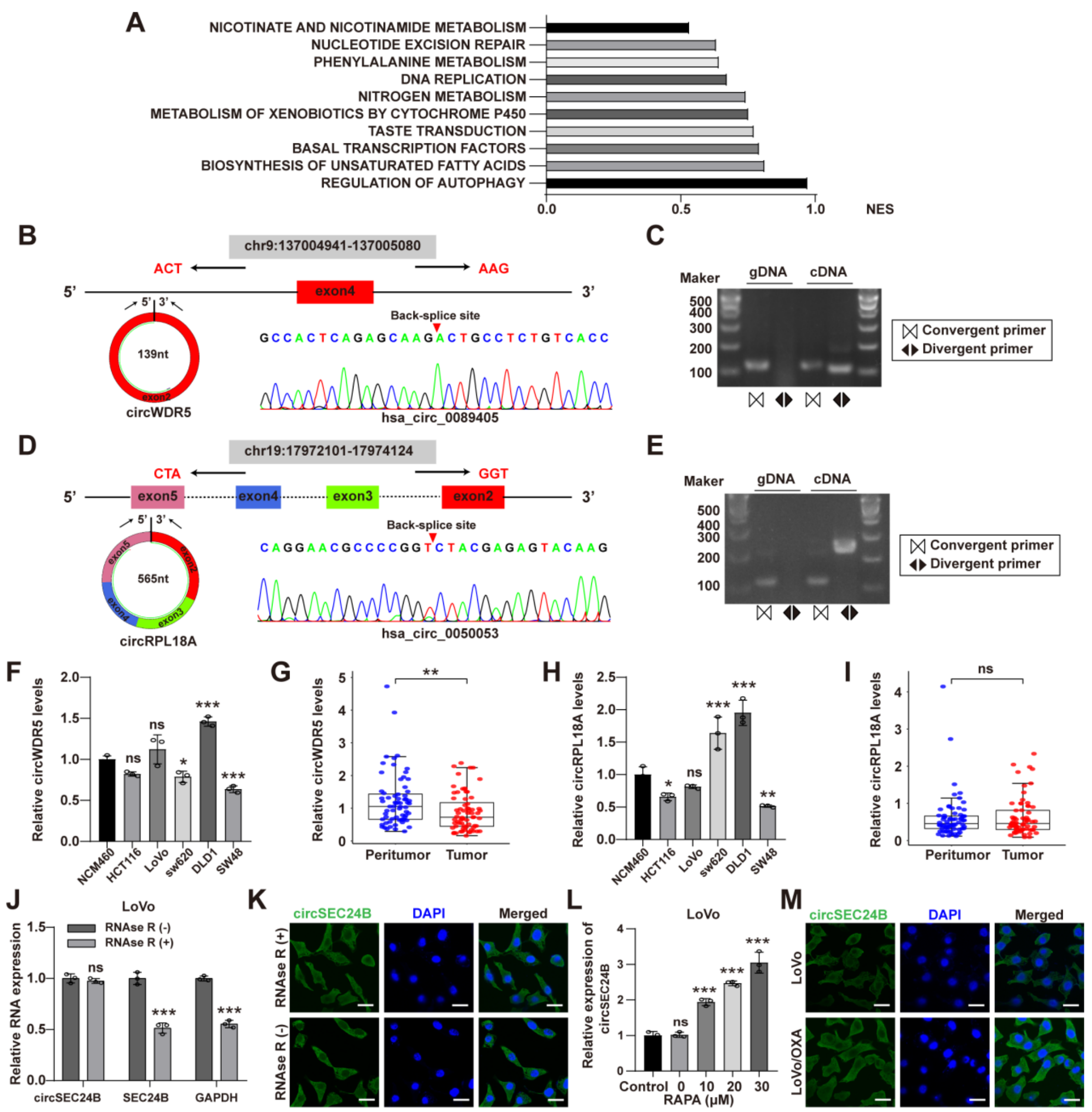

Fig. S1. Characterization of hsa_circ_0089405 and hsa_circ_0050053. **(A)** Gene set enrichment analysis (GSEA) with gene expression matrix of OXA-treated and untreated HCT116 cells revealed the top 10 pathways associated with the resistant cells. (B) The genomic locus of hsa_circ_0089405 (circWDR5). (C) RT-PCR assay with cDNA and genomic DNA by using hsa_circ_0089405 divergent and convergent primers. (D) The genomic locus of hsa_circ_0089405 (circRPL18A). (E) RT-PCR assay by using cDNA and genomic DNA with hsa_circ_0050053

divergent and convergent primers. Arrows stand for divergent primers that target its genome region. (F) RT-qPCR assays for circWDR5 with the NCM460 as well as the five CRC cell lines. (G) RT-qPCR assays for circWDR5 by CRC tissue as well as normal tissue samples. (H) RT-qPCR assays for circRPL18A by the NCM460 as well as the five CRC cell lines. (I) RT-qPCR assays for circRPL18A via CRC tissue as well as normal tissue samples. (J) RT-qPCR assay for circSEC24B, SEC24B and GAPDH with or without RNAse R treatment in LoVo cells. (K) RNA-FISH for circSEC24B with or without treatment of RNAse R in LoVo cells, DAPI (blue) was applied for nuclei staining. (L) RT-qPCR assay for circSEC24B expression by using different dose of RAPA in LoVo cell line. (M) RNA-FISH assay for circSEC24B in LoVo and LoVo/OXA cell lines.

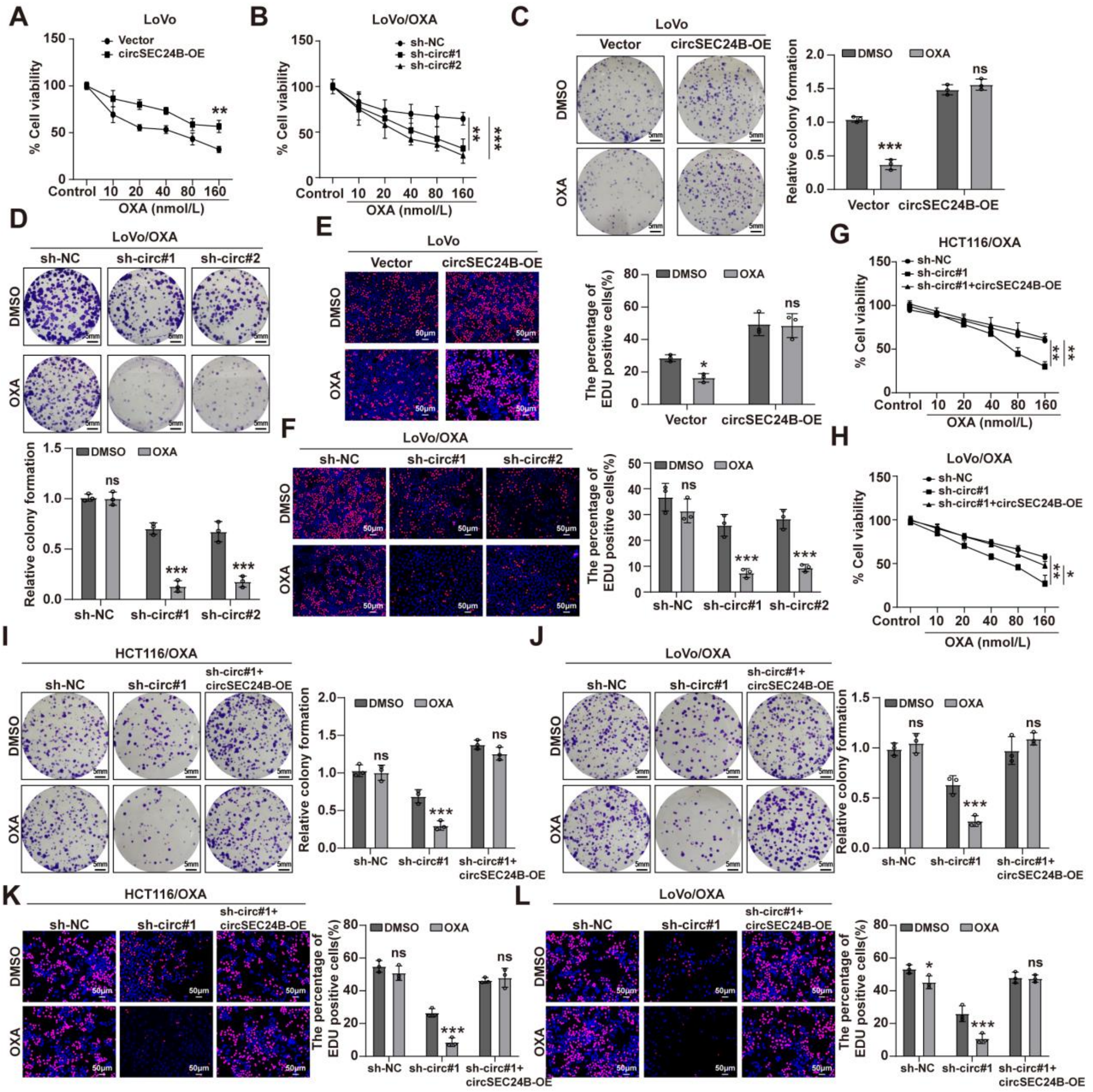

Fig. S2. CircSEC24B promoted CRC cell proliferation and OXA resistance. (A-F) The effect of circSEC24B knockdown or overexpression on cell viability and proliferation ability were assessed by CCK-8 (A, B), colony formation (C, D) and EDU assays (E, F) in the LoVo and LoVo/OXA cell line. (G-L) The effect of circSEC24B knockdown and/or overexpression on cell viability and proliferation ability were assessed by CCK-8 (G, H), colony formation (I, J) and EDU assays (K, L) in the HCT116/OXA and LoVo/OXA cell line.

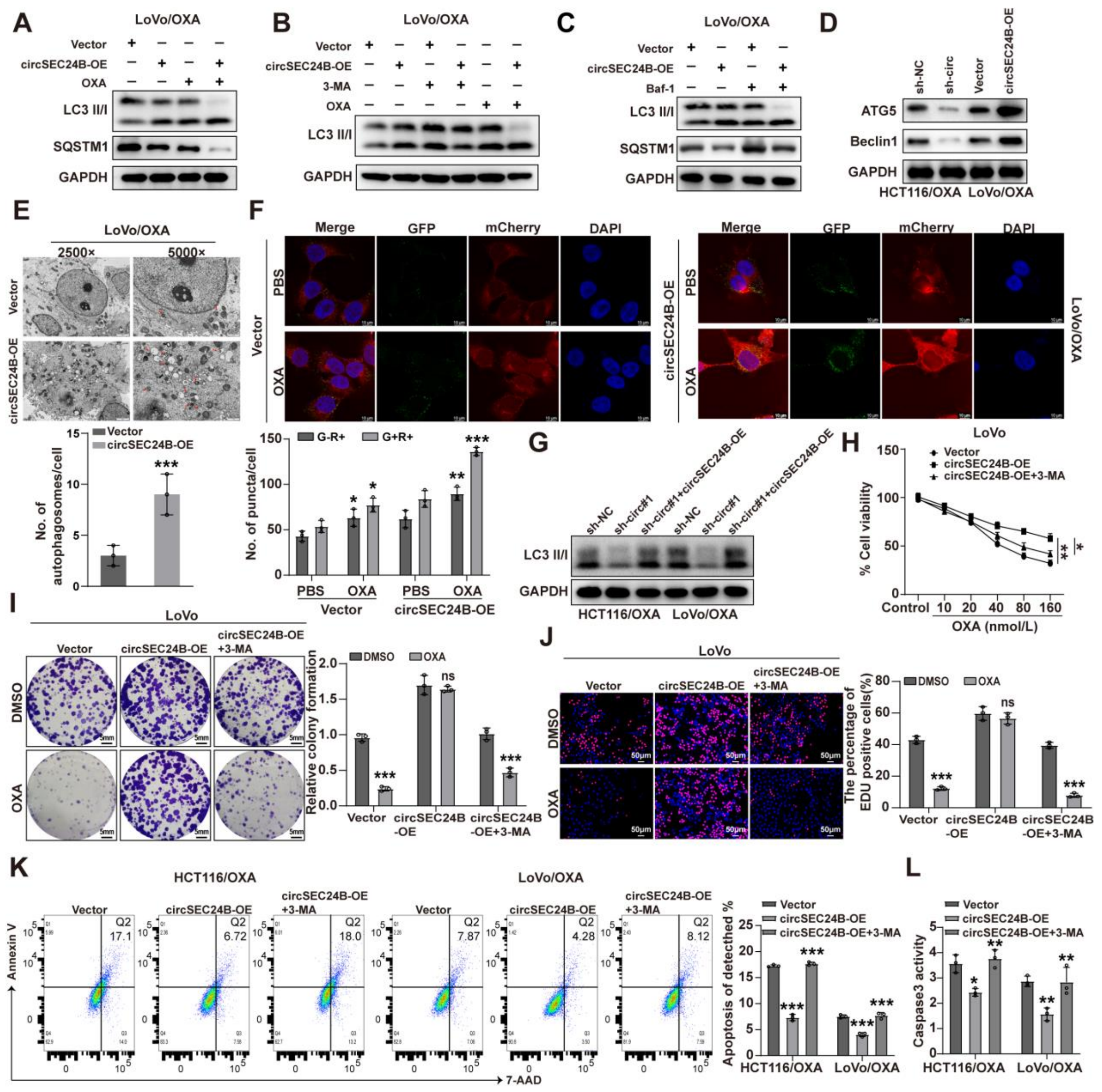

Fig. S3. CircSEC24B elevated the autophagy level of LoVo/OXA cells. (A) Western blotting assay for the expression levels of LC3-II and p62 with or without OXA treatment in the NC and circSEC24B-overexpression transfected LoVo/OXA cell line. (B) Western blotting assay for the expression levels of LC3-II with or without OXA, 3-MA treatment in NC or circSEC24B-overexpression transfected LoVo/OXA cells. (C) Western blotting assay for the expression levels of LC3-II and p62, Baf-1 treatment in NC or circSEC24B-overexpression transfected LoVo/OXA cells. (D) Western blotting assay for the expression levels of ATG5 and Beclin1 with

circSEC24B overexpression or knockdown transfected HCT116/OXA and LoVo/OXA cells. (E) Representative TEM images of the circSEC24B-overexpression transfected in the LoVo/OXA cell line. The red arrows stand for the autophagosomes in the cytoplasm. The graph below showed the numbers of autophagosomes in the cytoplasm. (F) Representative autophagy puncta images of the NC and circSEC24B-transfected LoVo/OXA cells with or without OXA treatment. (G) The influence of knockdown and overexpression of circSEC24B on LC3-II was evaluated. (H-J) The effects of circSEC24B-transfected LoVo cells with or without OXA and 3-MA treatment on cell viability and proliferation ability were assessed by CCK-8 (H), colony formation (I) and EDU assays (J). (K-L) Flow cytometry analysis (FACS) (K) and caspase 3 activity assay (L) were applied to measure the anoikis rate in circSEC24B-transfected LoVo/OXA and HCT116/OXA cells with or without 3-MA treatment.

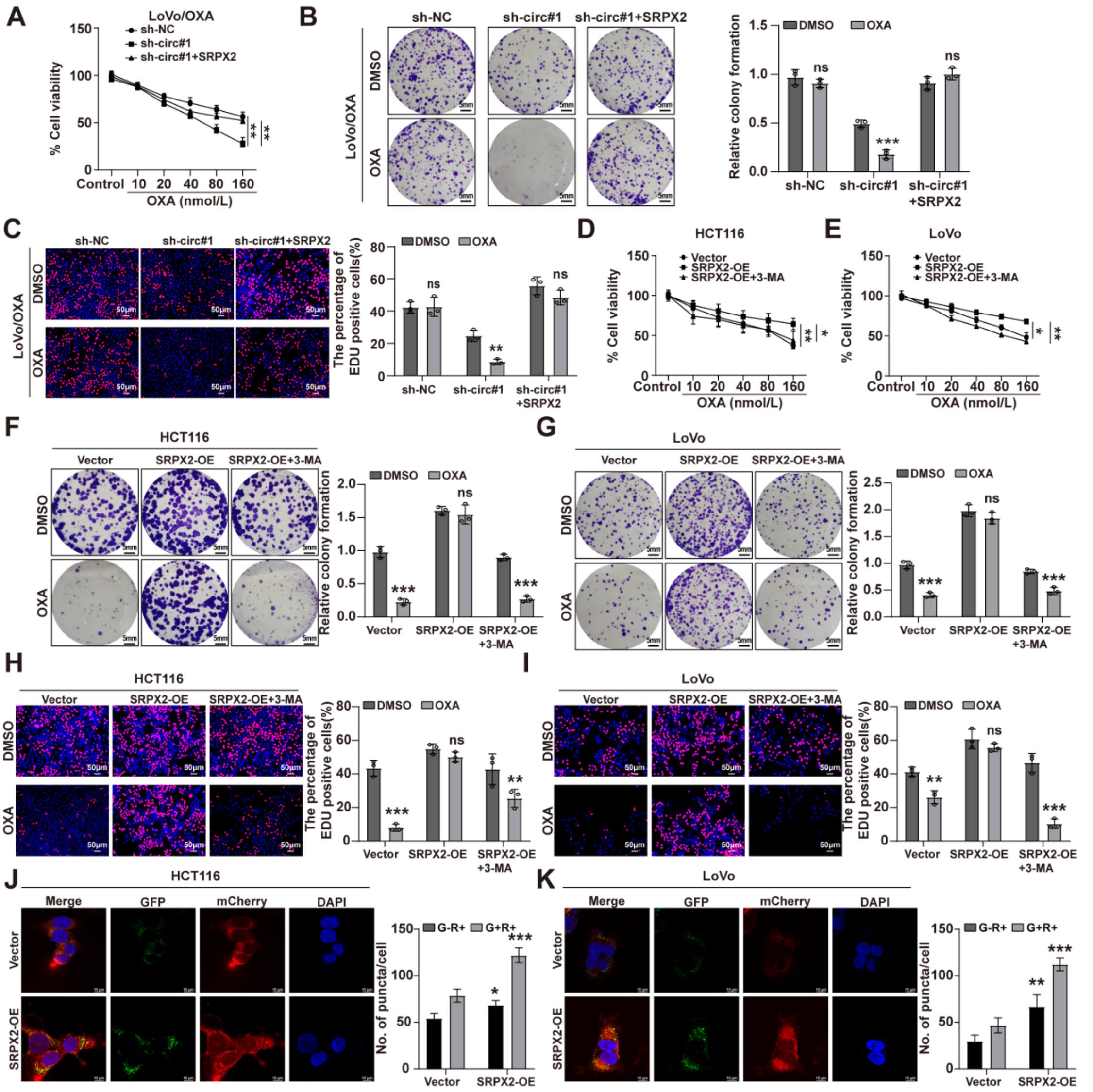

Fig. S4. CircSEC24B promoted CRC proliferative capacity via regulating the protein stability of SRPX2. (A-C) The effect of circSEC24B knockdown and/or SRPX2 overexpression on cell viability and proliferation ability were assessed by CCK-8 (A), colony formation (B) and EDU assays (C) in the LoVo/OXA cell line. (D-F) The effect of SRPX2 overexpression and 3-MA on cell viability and proliferation ability were assessed by CCK-8 (D, E), colony formation (F, G) and EDU assays (H, I) in the HCT116 and LoVo cell line. (J, K) The effect of SRPX2

overexpression on autophagy puncta were assessed by immunofluorescence assay in the HCT116 and LoVo cell line.

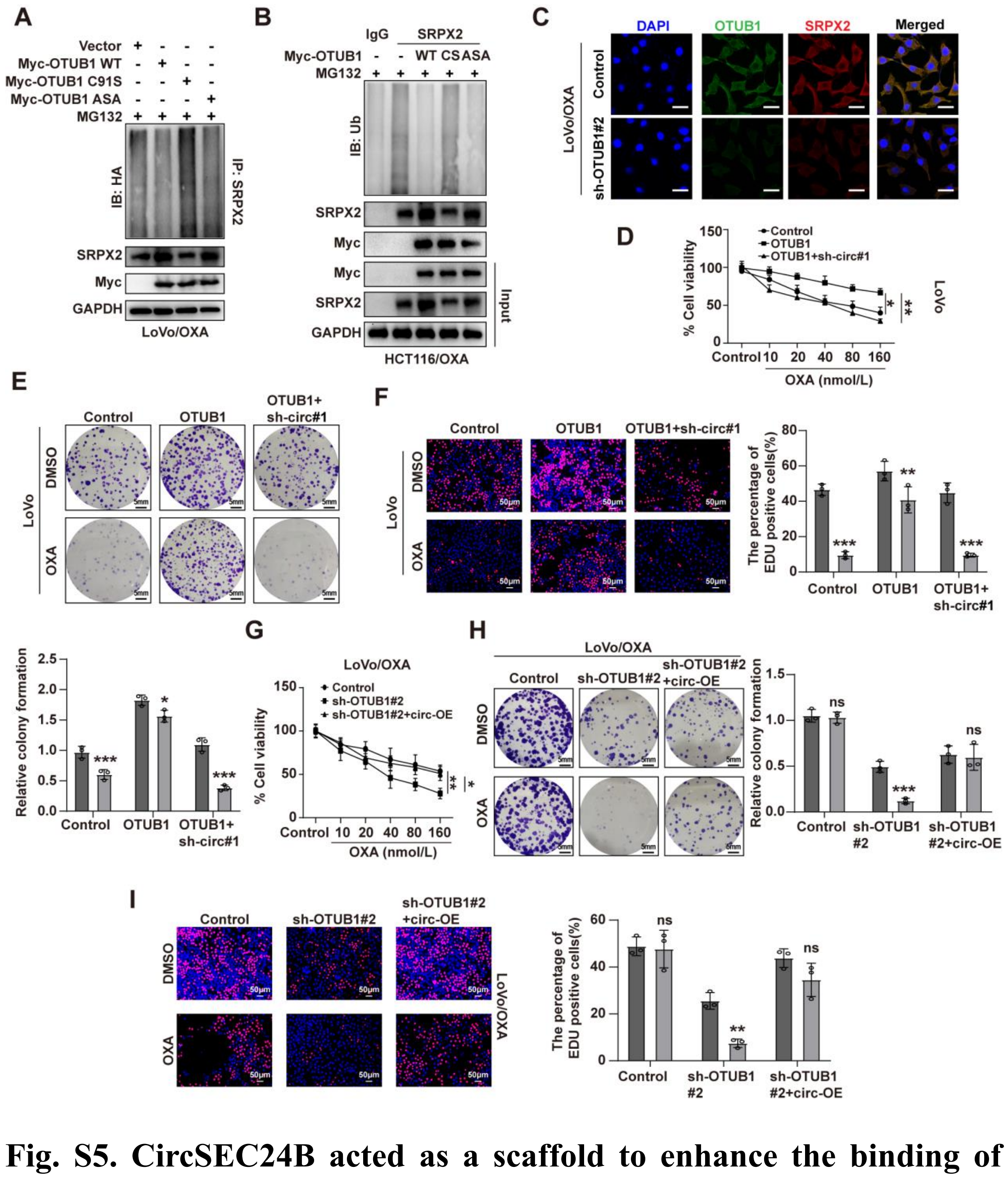

Fig. S5. CircSEC24B acted as a scaffold to enhance the binding of OTUB1 proteins with SRPX2. (A) CRC cells were transfected with plasmids harboring Myc-OTUB1 WT and each single mutant of Myc-

OTUB1 (C91S and ASA) in the presence of Flag-SRPX2, HA-Ub and MG132. The ubiquitination of endogenous SRPX2 was detected using the ubiquitination assay. (B) The effect of Myc-OTUB1 WT and each single mutant of Myc-OTUB1 on the ubiquitination level and protein level of SRPX2. (C) The effect of OTUB1 knockdown on the expression of SRPX2 by immunofluorescence assay in the LoVo/OXA cells. (D-F) The effect of OTUB1 overexpression and/or circSEC24B knockdown on cell viability and proliferation ability were evaluated by CCK-8 (D), colony formation (E) and EDU assays (F) in the LoVo cells treat with OXA or DMSO. (G-I) The effect of OTUB1 knockdown and/or circSEC24B overexpression on cell viability and proliferation ability were evaluated via CCK-8 (G), colony formation (H) and EDU assays (I) in the LoVo/OXA cells treated with OXA or PBS.
